# Loss of RET Promotes Mesenchymal Identity in Neuroblastoma Cells

**DOI:** 10.3390/cancers13081909

**Published:** 2021-04-15

**Authors:** Joachim T. Siaw, Jonatan L. Gabre, Ezgi Uçkun, Marc Vigny, Wancun Zhang, Jimmy Van den Eynden, Bengt Hallberg, Ruth H. Palmer, Jikui Guan

**Affiliations:** 1 Department of Medical Biochemistry and Cell Biology, Institute of Biomedicine, Sahlgrenska Academy, University of Gothenburg, SE-40530 Gothenburg, Sweden; joachim.siaw@gu.se (J.T.S.); jonatan.gabre@gu.se (J.L.G.); ezgi.uckun@gu.se (E.U.); Bengt.Hallberg@gu.se (B.H.); ruth.palmer@gu.se (R.H.P.); 2 Anatomy and Embryology Unit, Department of Human Structure and Repair, Ghent University, 9000 Ghent, Belgium; jimmy.vandeneynden@ugent.be; 3 Université Pierre et Marie Curie, UPMC, INSERM UMRS-839, 75005 Paris, France; marc.vigny@imserm.fr; 4 Department of Pediatric Oncology Surgery, Children’s Hospital Affiliated to Zhengzhou University, Zhengzhou 450018, China; zhangwancun@126.com

**Keywords:** ALK, ALKAL2, EMT, neural differentiation, adrenergic, retinoic acid

## Abstract

**Simple Summary:**

The anaplastic lymphoma kinase (ALK) and rearranged during transfection (RET) receptor tyrosine kinases (RTKs) are expressed in both the developing neural crest and the pediatric cancer neuroblastoma. Moreover, ALK is mutated in approximately 10% of neuroblastomas. Here, we investigated ALK and RET in neuroblastoma, with the aim of better understanding their respective contributions. Using neuroblastoma cell lines, we show that ALK modulates RET signaling at the level of RET phosphorylation, as well as at the level of transcription. Using CRISPR/Cas9, we generated RET knockout neuroblastoma cell lines and performed a multi-omics approach, combining RNA-Seq and proteomics to characterize the effect of deleting RET in a neuroblastoma context. Remarkably, we could show that loss of RET results in a striking epithelial-to-mesenchymal transition (EMT) phenotype, and we provide evidence that RET activity suppresses the mesenchymal phenotype in neuroblastoma.

**Abstract:**

Aberrant activation of anaplastic lymphoma kinase (ALK) drives neuroblastoma (NB). Previous work identified the RET receptor tyrosine kinase (RTK) as a downstream target of ALK activity in NB models. We show here that ALK activation in response to ALKAL2 ligand results in the rapid phosphorylation of RET in NB cells, providing additional insight into the contribution of RET to the ALK-driven gene signature in NB. To further address the role of RET in NB, *RET* knockout (KO) SK-N-AS cells were generated by CRISPR/Cas9 genome engineering. Gene expression analysis of *RET* KO NB cells identified a reprogramming of NB cells to a mesenchymal (MES) phenotype that was characterized by increased migration and upregulation of the AXL and MNNG HOS transforming gene (MET) RTKs, as well as integrins and extracellular matrix components. Strikingly, the upregulation of AXL in the absence of RET reflects the development timeline observed in the neural crest as progenitor cells undergo differentiation during embryonic development. Together, these findings suggest that a MES phenotype is promoted in mesenchymal NB cells in the absence of RET, reflective of a less differentiated developmental status.

## 1. Introduction

Neuroblastoma (NB) is a cancer that predominantly arises in young children. It is regarded as a disorder of imbalanced gene dosage due to numerous somatic chromosomal lesions, including structural variations and copy number alterations that result in aberrant cell signaling during development [1,2]. Mutation of the anaplastic lymphoma kinase (*ALK*) receptor tyrosine kinase (RTK) represents one of the verified oncogenes in NB [3,4,5,6,7,8,9,10]. ALK function in NB has been investigated in cell lines and other model systems, such as mouse, Drosophila and zebrafish [3,11]. Activation of ALK alone, by point mutation, amplification, or overexpression of the ALKAL ligand, does not appear to be sufficient to drive NB, and either the presence of an additional oncoprotein, such as v-myc avian myelocytomatosis viral oncogene neuroblastoma derived homolog (MYCN), or the loss of critical tumor suppressors is thought to be critical for NB to progress [10,12,13,14,15,16,17,18,19,20,21,22]

Rearranged during transfection (RET) is another RTK that is expressed in the developing neural crest and NB. RET is activated by the glial cell line-derived neurotrophic factor (GDNF) family ligands (GLFs) [23]. RET activation is complex, with GLFs first binding the GDNF family coreceptor members, which subsequently interact with and activate RET, activating downstream signaling [24,25,26]. Moreover, RET activation can be modulated by other membrane receptors, such as tropomyosin-related kinase B (TrkB) [27,28]. While mice lacking the ALK RTK are viable [29,30,31], mice lacking the RET RTK are deprived of enteric neurons in the digestive tract and exhibit defects in kidney organogenesis, resulting in death at birth [32,33]. It has been shown that RET function is important for the survival and differentiation of the nervous system [23]. Both gain- and loss-of-function mutations of RET are found in human disease, including cancers with neuroendocrine origin and the intestinal syndrome known as Hirschsprung disease [23]. Oncogenic RET results from either activating mutations or chromosomal rearrangements that produce RET fusion oncogenes, and these are found in a range of cancers, including papillary thyroid carcinoma, medullary thyroid carcinoma, and the multiple endocrine neoplasia types 2A and 2B (MENA2A and MENA2B) [23]. No mutations of *RET* have been described in NB [9,10,34,35]; however, RET expression has been reported to be regulated by ALK in both mouse models and NB cell lines [21,24,36,37]. Furthermore, elevated RET expression is observed in ALK-mutated primary NB tumor samples [24]. The role of RET in differentiation in NB is unclear, although it is known that RET is expressed in tissues originating from the neural crest [23,38]. Furthermore, RET expression increases in NB cell lines in response to retinoic acid (RA) induction, and activated RET leads to NB cell differentiation [39,40,41,42]. RA-induced differentiation of NB cells leads to increased expression of both TrkA and RET messenger RNA (mRNA), and reports indicate that both RTKs cooperate in the differentiation process [28,43,44].

Recent studies have demonstrated that primary NBs and NB cell lines may consist of two major cell types of different differentiation states, namely, neural crest cell (NCC)-like mesenchymal (MES) and more committed adrenergic (ADRN) cells [45]. MES cells are more resistant to therapy, such as chemotherapy, and are found to be enriched in relapsed NB tumors [45]. MES signature genes include Yes-associated protein 1 (*YAP1*), WW domain–containing transcription regulator 1 (*WWTR1*), fibronectin 1 (*FN1*), Hes family bHLH transcription factor 1 (*HES1*), and snail family transcriptional repressor 2 (*SNAI2*) among others [45]. YAP1 and transcriptional coactivator with PDZ-binding motif (TAZ, encoded by *WWTR1*) are transcriptional coactivators which act as the main downstream effectors of the Hippo pathway. These transcription factors were shown to be activated by an alternative Wnt signaling pathway [46]. It has been suggested that the Wnt signaling pathway acting through the transcription factors paired related homeobox 1 (PRRX1) and YAP/TAZ, serves as the major mediator of the regulatory networks that drive MES/NCC-like identities of NB cells [47].

In this work, we describe activation of RET phosphorylation in response to activation of the ALK RTK. We also confirm that ALK signaling regulates RET transcription and that this requires the RAS/MAPK pathway activity. To further investigate RET function in NB cells, we generated *RET* knockout (KO) SK-N-AS cell lines by CRISPR/Cas9 genome modification, which exhibited strong epithelial-to-mesenchymal transition (EMT) RNA and protein signatures. We further investigated the requirement of RET in NB cell differentiation using two RET inhibitors, LOXO292 and BLU667, showing that RET activity is important for RA-induced NB cell differentiation in a context-dependent manner.

## 2. Results

### 2.1. Stimulation of ALK in NB Cells Leads to RET Receptor Phosphorylation

**Figure 1:**
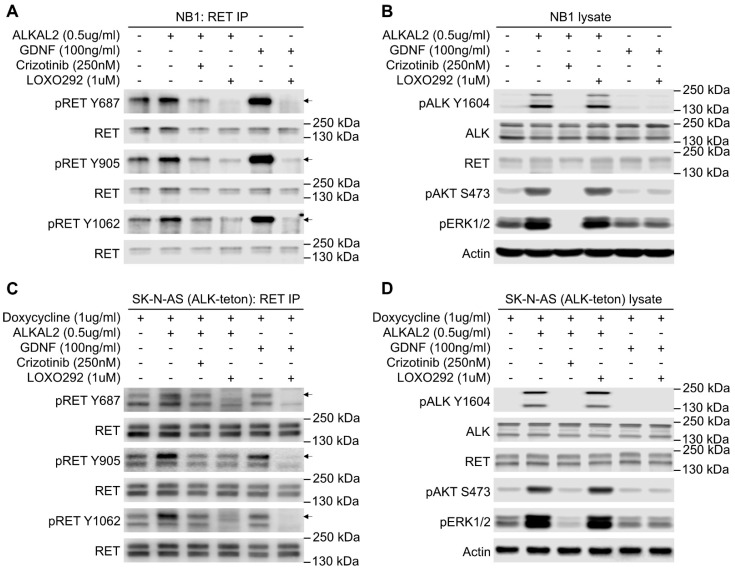
Anaplastic lymphoma kinase (ALK) activation leads to rearranged during transfection (RET) phosphorylation. (**A**) Western blot analysis of RET immunoprecipitates (IPs) from NB1 cells treated with combinations of either ALKAL2, glial cell line-derived neurotrophic factor (GDNF), crizotinib, or LOXO292 as indicated. Phospho-RET antibodies were used to indicate RET activation, and blots reprobed with RET antibody were used as control. (**B**) Corresponding NB1 cell lysates were blotted to show activation of ALK and downstream signaling. Total ALK, RET, and actin were used as loading controls. (**C**) Western blot analysis of RET immunoprecipitated from ALK-inducible SK-N-AS cells which express ALK upon addition of doxycycline. Cells were treated as in (**A**), with combinations of ALKAL2, GDNF, crizotinib, or LOXO292 as indicated. Phospho-RET antibodies were used to indicate RET activation, and blots reprobed with RET antibody were used as control. (**D**) Corresponding ALK-inducible SK-N-AS (ALK-teton) cell lysates were blotted to show activation of ALK and downstream signaling. Total ALK, RET, and actin were used as loading controls. Arrows indicate a slower migrating form of RET that likely corresponds to surface RET protein.

**Figure 2:**
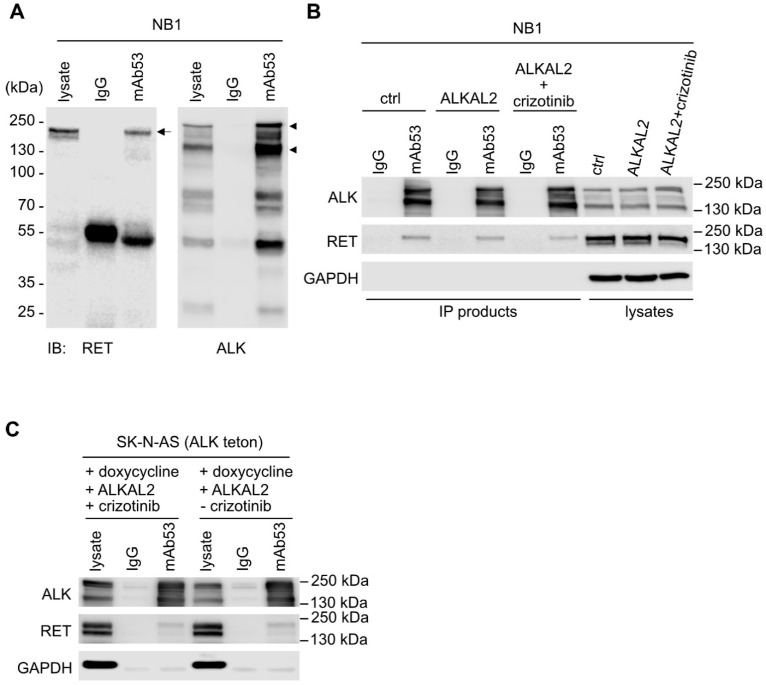
ALK interacts with RET. (**A**) Western blot analysis of ALK immunoprecipitation from NB1 cells, showing the presence of RET in ALK IP products. (**B**) The presence of RET in ALK IPs is not affected by ALK activity. Arrows indicate a slower-migrating form of RET that likely corresponds to surface RET protein; arrowheads highlight the 220 kDa and 140 kDa bands which correspond to full-length and cleaved ALK. House-keeping protein glyceraldehyde-3-phosphate dehydrogenase (GAPDH) was used as control. (**C**) Co-immunoprecipitation of RET with ALK in ALK-inducible SK-N-AS (ALK-teton) cells upon addition of doxycycline, independent of ALK activation.

We previously showed that inhibition of ALK with either crizotinib or lorlatinib results in decreased *RET* mRNA and protein levels in ALK-driven NB cell lines [36]. This is in line with the findings of Lambertz et al. and Cazes et al. [21,24], who observed increased *Ret* mRNA in ALK/MYCN versus MYCN tumor models, and could show that *ALK*-amplified or mutant cell lines, as well as tumors, were sensitive to the vandetanib tyrosine kinase inhibitor (TKI), which inhibits RET among other kinase targets. Although RET phosphorylation has been reported in NB cell lines [24,44] and to be modulated by ALK inhibition [36], studies to date have reported the role of ALK in regulating *RET* mRNA expression [21,24,36]. We were, therefore, surprised to observe activation of the RET receptor at 10 min, as measured by pRET-Y687, -Y905, and -Y1062 (Figure 1A,B) in response to stimulation of NB1 cells with the ALK ligand, ALKAL2. NB1 cells were chosen as they express nonmutated ALK that can be further stimulated by ALKAL2 [14,15,48]. As expected, increased pRET-Y687, -Y905, and -Y1062 was also observed when the RET ligand GDNF was employed to activate RET, and this was sensitive to the RET TKI LOXO292 (Figure 1A). This activation of RET on ALKAL2 stimulation was not seen in the presence of the ALK TKI crizotinib (Figure 1A). Phosphorylation of RET on Y687, Y905, and Y1062 in response to ALK activation was also confirmed in an additional NB cell line, SK-N-AS (ALK-teton), which was modified to express wildtype ALK in response to doxycycline (Figure 1C,D; Figure S1B). SK-N-AS was chosen as it does not express detectable levels of ALK protein, allowing ALK expression levels and activation to be controlled. Once again, this ALKAL2-mediated phosphorylation of RET was sensitive to crizotinib (Figure 1C). RET expressed in SK-N-AS (ALK-teton) cells was also activated in response to GDNF, and this was abrogated in the presence of LOXO292. While ALK activation resulted in phosphorylation of RET, we did not observe a reciprocal activation of ALK in response to GDNF (Figure 1B,D). We also note that both pRET-Y687 and pRET-Y905 antibodies strongly cross-react with activated ALK, necessitating a careful analysis since ALK and RET are of a similar, although not identical, molecular weight (Figure S1A). Interestingly, we were unable to observe activation of downstream AKT and ERK signaling pathways in either NB1 or SK-N-AS (ALK-teton) cells in response to GDNF (Figure 1). We further tested the efficacy of GDNF in stimulation of downstream signaling in CLB-GAR, CLB-BAR, and CLB-GE cells, observing phosphorylation of AKT and ERK1/2 in CLB-GAR cells, which is inhibited in the presence of the RET inhibitor LOXO202 (Figure S2). Given the observed activation of RET in response to ALKAL2, we asked whether ALK and RET physically interact. We identified the RET receptor in ALK immunoprecipitated complexes, and this interaction was apparently independent of ALK activation, since similar levels of RET were immunoprecipitated in the presence of either ALKAL2 or crizotinib (Figure 2A–C). Taken together, our observations suggest that ALK and RET physically interact and that RET can be phosphorylated upon ALK activation.

### 2.2. RAS/MAPK Signaling but Not PI3K/AKT Regulates RET Transcription

**Figure 3:**
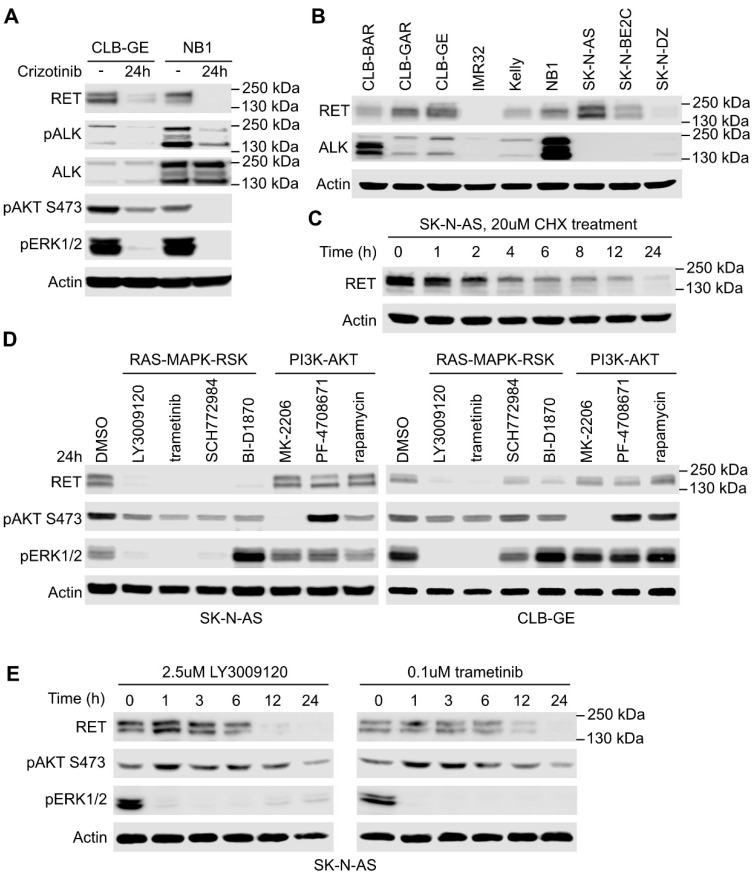
RAS/MAPK but not PI3K/AKT signaling pathway regulates RET expression. (**A**) Western blot analysis of the effect of ALK inhibition on RET expression. Two ALK-driven neuroblastoma (NB) cell lines, CLB-GE (ALK-F1174V) and NB1 (ALK-amplified), were treated with crizotinib for 24 h and total lysate was analyzed for proteins as indicated; actin was used as loading control. (**B**) RET and ALK protein expression profile in a panel of NB cell lines. (**C**) Western blot analysis of RET protein levels in response to cycloheximide (CHX) treatment in SK-N-AS cells. (**D**) RET protein levels in SK-N-AS and CLB-GE NB cells in response to inhibition of RAS/MAPK/RSK and PI3K/AKT/mTOR signaling pathways. Cells treated with solvent dimethyl sulfoxide (DMSO) were used as control. (**E**) Western blot analysis of RET protein levels over time in response to LY3009120 and trametinib treatment in SK-N-AS cells.

Inhibition of ALK with crizotinib for 24 h results in decreased RET protein levels in CLB-GE and NB1 cells (Figure 3A). To investigate the regulation of RET expression, we first assessed RET protein levels in a panel of NB cell lines, identifying SK-N-AS as an NB cell line with easily detectable RET expression (Figure 3B). To address whether RET levels are maintained in SK-N-AS cells transcriptionally, we examined RET protein levels in response to cycloheximide (CHX), which blocks protein translation. As expected, CHX addition led to a time-dependent decrease in RET protein with a half-life between 2 and 4 h (Figure 3C). To further dissect which downstream signaling pathways are important for RET expression, we focused on RAS/MAPK/RSK and PI3K/AKT/mTOR pathways. SK-N-AS and CLB-GE cells were incubated with a range of inhibitors targeting both RAS/MAPK/RSK and PI3K/AKT/mTOR signaling pathways (Figure 3D). Inhibition of RAS/MAPK signaling, employing the pan-RAF inhibitor LY3009120, the MEK1/2 inhibitor trametinib, the ERK1/2 inhibitor SCH772984, or the RSK1/2/3/4 inhibitor BI-D1870, led to a loss of RET protein over time that was clearly visible at 24 h (Figure 3D,E). In contrast, RET protein levels were not significantly altered at 24 h in cells treated with inhibitors targeting the PI3K/AKT pathway (Figure 3D), suggesting that oncogenic signaling through the RAS/MAPK pathway is important to maintain RET levels in NB cells in agreement with previous findings [37,49].

### 2.3. Loss of RET Leads to Phenotypic Changes and Reduced Proliferation

**Figure 4:**
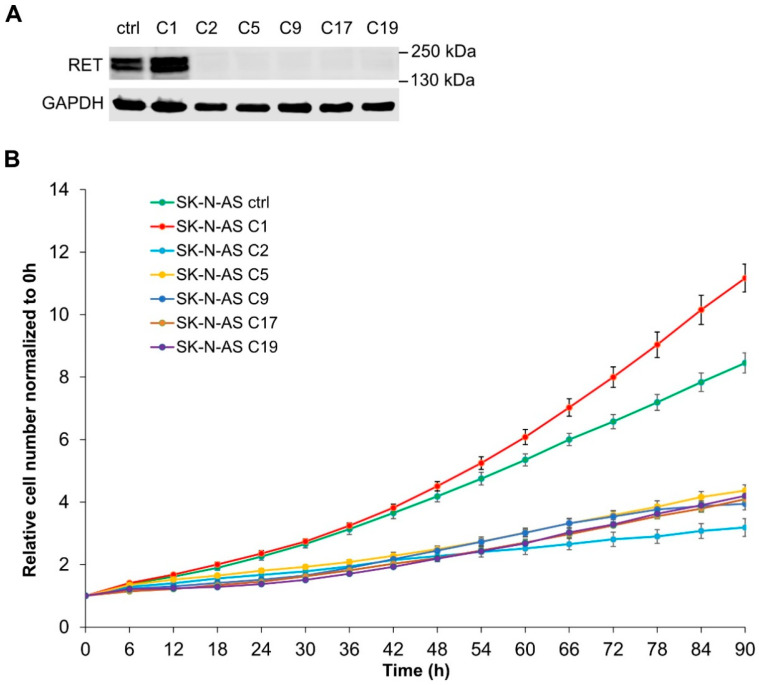

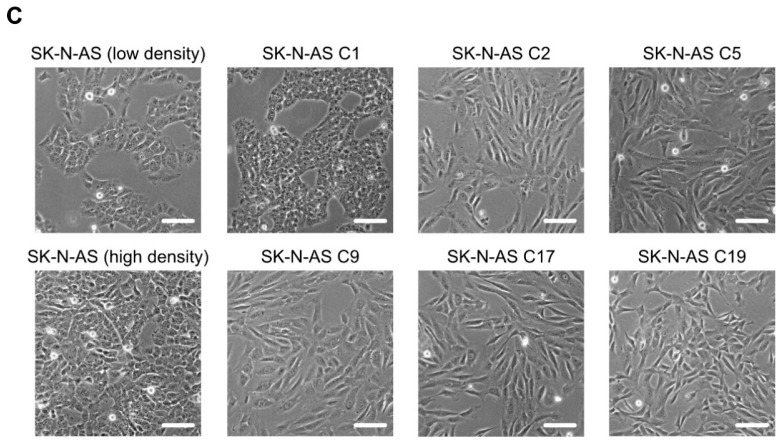
Characterization of *RET* knockout (KO) SK-N-AS cells. (**A**) Western blot analysis of RET expression in the parental (ctrl) and multiple clones of *RET* CRISPR/Cas9 engineered SK-N-AS cells. GAPDH was used as loading control. (**B**) Cell proliferation analysis of *RET* CRISPR/Cas9 engineered SK-N-AS cells with Incucyte S3 Live-Cell Analysis System and Nuclight Rapid Red Dye for live-cell nuclear labeling. (**C**) Bright-field microscopy images of indicated cells indicating morphological differences observed in *RET* CRISPR/Cas9 engineered SK-N-AS cells (scale bar = 50 μm).

To better understand the role of RET in NB cell lines, we generated *RET* KO lines by CRISPR/Cas9 genome modification. As we previously generated inducible ALK lines in the SK-N-AS cell lines, we chose to remove *RET* with CRISPR/Cas9 in this cell line. SK-N-AS cells lacking *RET* were generated and confirmed at the genomic level as *RET* KO. As expected, these *RET* KO clones (SK-N-AS C2, C5, C9, C17, and C19) lacked detectable RET protein expression, when compared with the parental control (Figure 4A). While screening for *RET* KO clones, we identified a cell clone that expressed a higher level of RET (SK-N-AS C1) compared with the parental line, which we included in our analyses. All SK-N-AS-derived cell lines (C1, C2, C5, C9, C17, and C19) were subjected to cell-line authentication that confirmed them as identical to the parental SK-N-AS cell lines. In addition, RNA-Seq analysis confirmed loss of *RET* mRNA in SK-N-AS C2, C5, C9, C17, and C19 *RET* KO clones, as well as identification of an genetic alteration in intron 1 of *RET* in the SK-N-AS C1 clone that exhibited increased RET protein levels (Table S2). We examined growth of SK-N-AS C2, C5, C9, C17, and C19 *RET* KO cell lines and noted that all SK-N-AS cells lacking RET exhibited decreased proliferation levels compared with the parental control (Figure 4B). Interestingly, an increased rate of growth was observed for the RET-overexpressing cell line SK-N-AS C1 as compared with the parental line (Figure 4B). During cell culture, we also noted a phenotypic change in SK-N-AS-derived cell lines lacking RET, with cells spreading more and exhibiting a mesenchymal morphology when compared to the parental cell line, whereas SK-N-AS C1 cells were smaller and rounder (Figure 4C). In addition, the appearance of multinucleated cells after a few passages was also observed in these SK-N-AS-derived *RET* KO cells, suggesting that there might be some deficiency in cell division (Figure S3) which is worthy of further analysis in the future. Given the striking reduction in cell growth and the morphological changes observed we decided to further investigate the SK-N-AS *RET* KO cells at the molecular level.

### 2.4. SK-N-AS RET KO NB Cells Display a Striking EMT Signature

**Figure 5:**
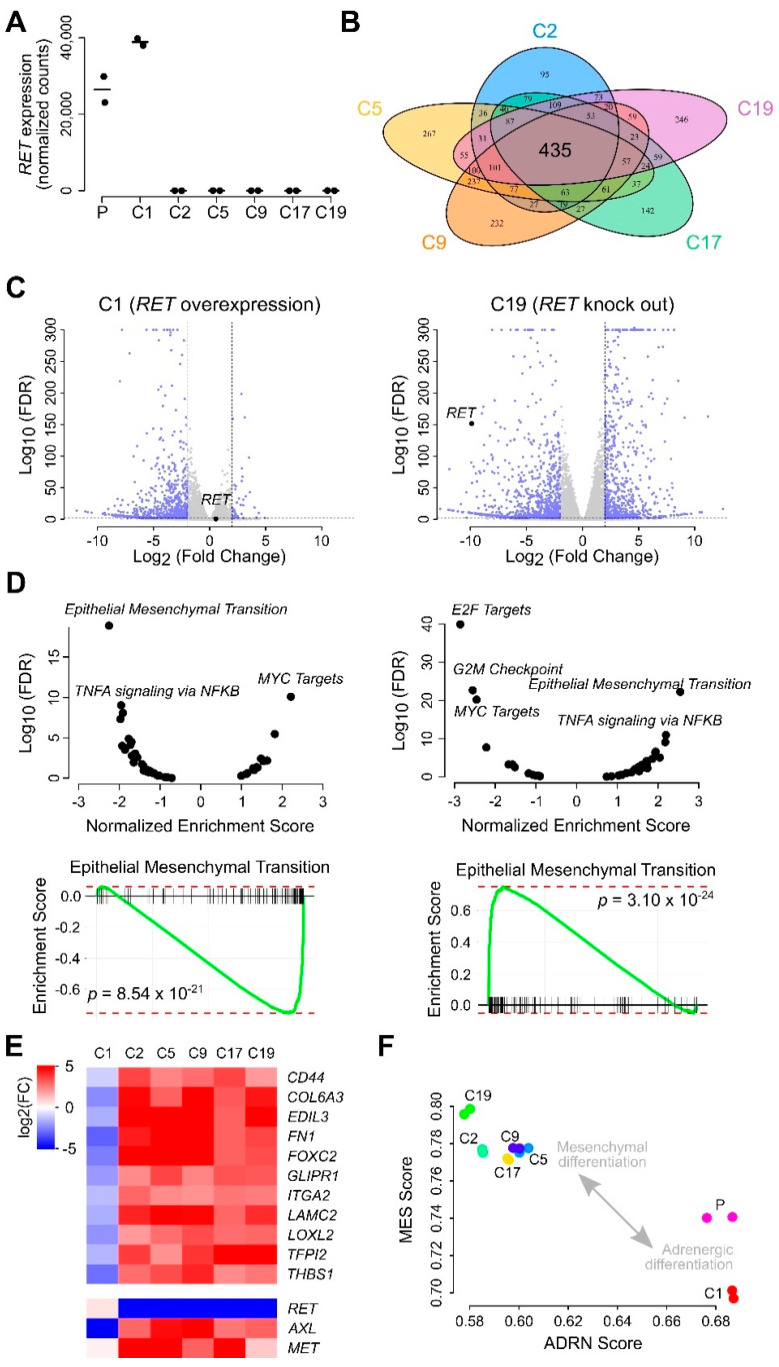
RNA-Seq differential gene expression analysis of *RET* KO NB cell lines. (**A**) Confirmation of *RET* KO in different clones. Horizontal lines indicate median values. P stands for parental cells. (**B**) Venn diagram showing the number of differentially expressed (DE) genes for the five *RET* KO clones as indicated. (**C**) Volcano plots showing DE for clone C1 (*RET* overexpression) and C19 (*RET* KO). Dashed lines indicate DE thresholds. Up-/downregulated genes indicated in blue. (**D**) Ranked gene set enrichment analysis (GSEA) results for C1 (left plots) and C19 (right plots). Upper panels show normalized enrichment scores and corresponding false discovery rate (FDR) values with labelling of most enriched gene sets. Lower panels represent running score plots for epithelial to mesenchymal transition. (**E**) Heatmap showing log2FC DE values for 11 EMT genes that were upregulated in all *RET* KO clones (top) and for *RET*, *AXL* and *MET* (bottom). (**F**) Mesenchymal (MES) and adrenergic (ADRN) signature scores for the different clones. All experiments were performed using two biological replicates in each condition. See Table S1 for detailed results.

To evaluate the overall transcriptomic response in the different *RET* KO clones, we performed RNA-Seq (Table S1). This analysis confirmed the absence of *RET* expression by clones C2, C5, C9, C17, and C19 on the one hand and *RET* overexpression by clone C1 on the other hand (Figure 5A). Compared to the control (parental) cell line, we observed a strong transcriptional response in *RET* KO clones, with 435 genes that were differentially expressed (DE) by all clones (DE thresholds log_2_fc ±2 at 1% false discovery rate (FDR); Figure 5B,C; Table S1). The upregulated genes included many extracellular matrix proteins (e.g., *FN1*, *COL20A1*, *COL13A1*, *COL6A3*, and *COL4A6*), as well as the *AXL* RTK, and they were strongly enriched for EMT, as exemplified for C19 in Figure 5D (*p* = 3.10 × 10^−24^). A highly similar EMT enrichment in *RET* KO cells was also observed at the protein level (Figure S4). Furthermore, we observed enrichment for tumor necrosis factor alpha (TNFα) signaling in the upregulated gene set, as well as *MYC* target genes in the downregulated gene set. Remarkably, a transcriptional downregulation was also observed in the *RET*-overexpressing clone C1. The gene set enrichment analysis of these genes mirrored the results from the *RET* KO clones, with EMT and TNFα enrichment for the downregulated and MYC target enrichment for the upregulated genes (Figure 5D,E).

Our results suggest a mesenchymal (MES) type of differentiation upon loss of RET. Therefore, we examined how these transcriptional responses were related to a published set of adrenergic (ADRN) and MES signature genes, using a rank percentile-based scoring method [45]. This analysis suggests a stronger MES identity of the *RET* KO clones (higher MES scores and lower ADRN scores, as compared to control) and an opposite ADRN differentiation in the *RET*-overexpressing clone (lower MES scores and higher ADRN scores as compared to control; Figure 5F). These results support an important role for RET in NB differentiation and show that downregulation of RET promotes a mesenchymal identity of NB cells.

### 2.5. RET KO Cells Display Enhanced Cell Migration

**Figure 6:**
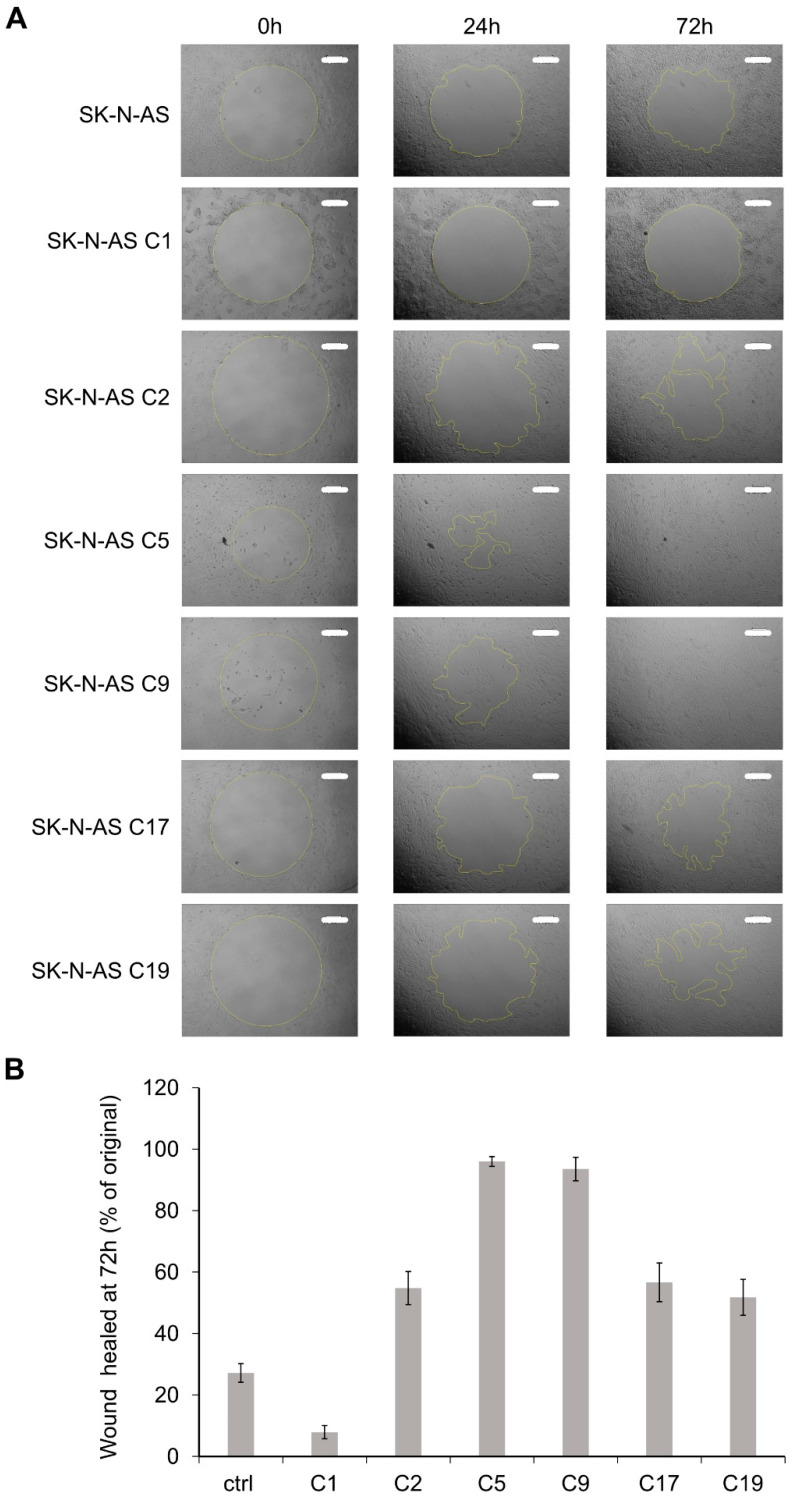
SK-N-AS *RET* KO cells display enhanced cell migration. (**A**) Phase-contrast microscopy images of indicated *RET* CRISPR/Cas9 engineered SK-N-AS cells at three time-points (0 h, 24 h, and 72 h) showing the migration difference with radius cell migration assay (scale bar = 300 μm). Irregular lines indicate the front edge of cell migration, and the cell migrating areas were quantified in (**B**) with ImageJ 1.53a. Values represent the mean ± SD from three independent experiments.

**Figure 7:**
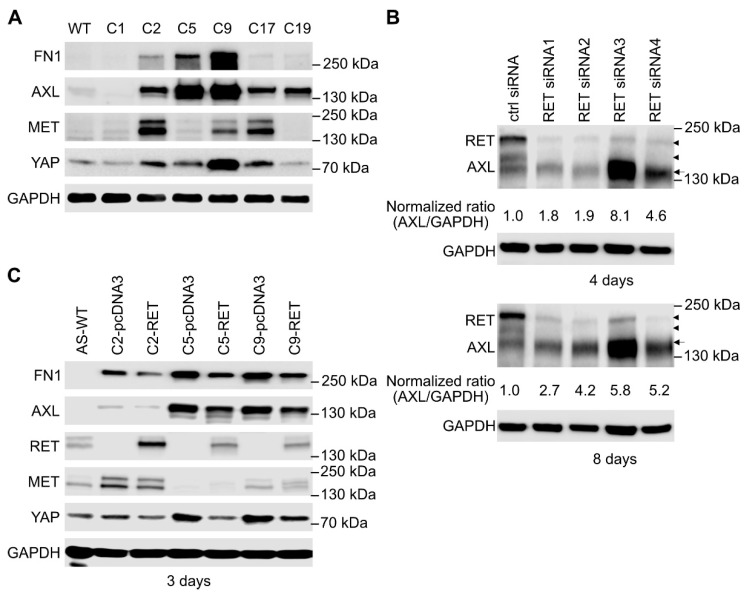
RET inhibits MES signature genes in SK-N-AS cells. (**A**) Western blot analysis of expression of selected MES signature proteins as indicated in *RET* CRISPR/Cas9 engineered SK-N-AS cells. (**B**) Knockdown of RET by small interfering RNA (siRNA) in SK-N-AS cells led to increased expression of AXL protein, whereas restored expression of RET in *RET* KO clones led to a trend in decreased expression of MES signature proteins (**C**). (**B**,**C**) Arrowheads indicate two bands of RET protein; arrow indicates AXL. Fibronectin 1 (FN1), MNNG HOS transforming gene (MET), Yes-associated protein (YAP).

Previous studies highlighted a role for EMT in increasing the motility and invasive capacity of tumor cells [50]. Moreover, the neural crest cells, from which NB arises, are highly migratory during development [51]. We, therefore, examined cell migration in the SK-N-AS *RET* KO cells. Employing a wound healing assay, we observed that NB cells lacking RET displayed increased cell motility compared to parental controls (Figure 6A, quantified in 6B). In keeping with this observation, we also noted increased levels of the AXL and MET RTKs in SK-N-AS cells lacking RET (Figure 5E and Figure 7A; Figure S4 and Table S1). Interestingly, although there were variable levels of increased migration in the different SK-N-AS *RET* KO clones, we noted that those clones that expressed most AXL were also those with the highest motility (Figure 6B). While upregulation of AXL was also observed when employing small interfering RNA (siRNA) targeting *RET* to the parental SK-N-AS cell line, this was variable and may reflect differences in response to loss of RET RNA with the siRNA approach, in contrast to long-term adaptation of cells to loss of the *RET* locus on CRISPR/Cas9 modification (Figure 7B). In addition, extracellular matrix proteins such as FN1 and Collagen 6A3 (COL6A3) were upregulated in *RET* KO cell lines (Figure 7A; Figure S4 and Table S1). Overexpression of RET in SK-N-AS *RET* KO clones led to a decrease in FN1, AXL, and MET to varying degrees (Figure 7C). In addition to AXL, we noted increased levels of the YAP1 transcription factor, which has been reported to promote EMT [52], in *RET* KO clones (Figure 7A; Figure S4 and Table S1).

### 2.6. Ret Expression Correlates with Differentiation in the Developing Sympathaticosystem

*Ret* expression has been suggested to be absent in nerve-associated Schwann cell precursor (SCP) cells at day E13.5 of mouse development. SCPs develop during embryogenesis to form adrenal chromaffin cells through an intermediate “bridge cell” stage [53]. These findings suggest that the enhanced mesenchymal identity we observed after *RET* KO might display similarities to SCPs and prompted us to investigate *RET* dynamics in the developing neural crest, exploiting the single-cell mouse embryonic database generated by Furlan and colleagues [53].

**Figure 8:**
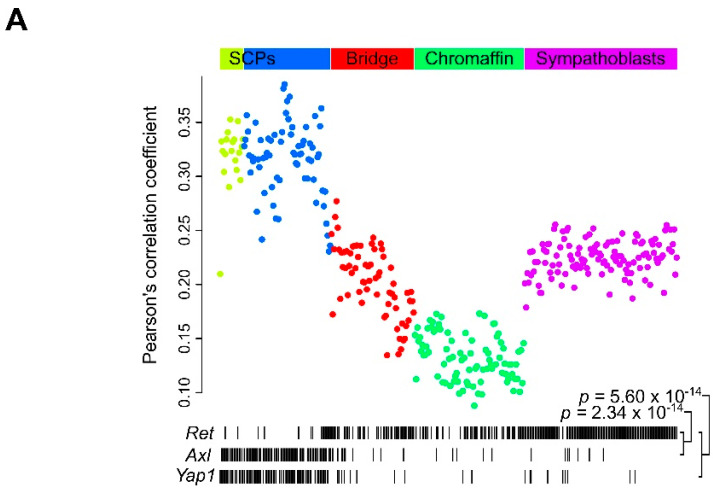

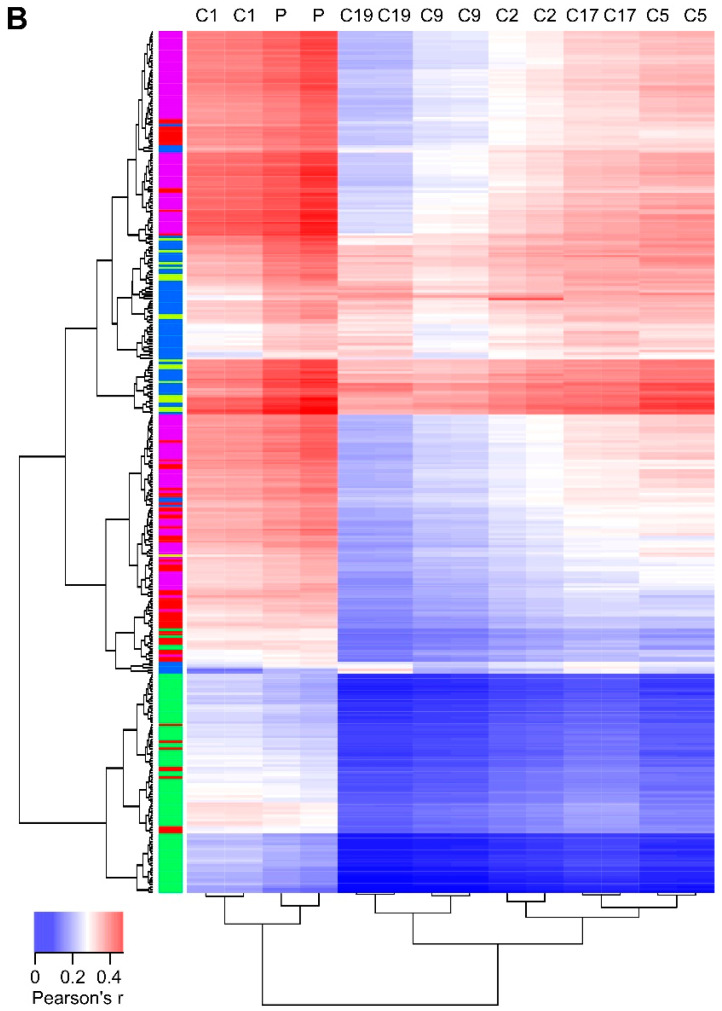
Neural crest single cell differentiation analysis. Single-cell gene expression data from Furlan et al. [53] (E13.5) were correlated to the expression of the clones used in this study. (**A**) Each dot represents one cell, with Pearson correlation coefficients with C19 *RET* KO clone shown on the *y*-axis and developmental timeline indicated by the *x*-axis. Cells are colored according to their type as indicated on top (as derived from Furlan et al.). The absence or presence of *Ret*, *Axl* and *Yap1* gene expression is shown below the plot with indication of *p*-values (Fisher’s exact test). (**B**) Heatmap showing Pearson correlation coefficients (key on bottom left) between each clone and single cell as indicated. Rows (cells) and columns (clones, P stands for parental cells) were hierarchically clustered with dendrograms shown above and on the left of the plot. Cell type indicated on the left of the plot by color bars. See panel (**A**) for color legend.

The AXL RTK has been connected to EMT, invasion, and metastasis in several cancers [54]. We observed that *AXL* was upregulated in all *RET* KO clones (log_2_FC = 2.66 or higher) and downregulated in the *RET*-overexpressing clone C1 (log_2_FC = −5.20; Table S1), while it had a near mutual exclusive expression pattern with *RET* (*p* = 2.34 × 10^−24^, Fisher’s exact test) with the majority of SCPs expressing *AXL* (85%), but rarely expressing *RET* (17%; Figure 8A). Interestingly, we also noted a similar pattern of mutual exclusivity (*p* = 5.60 × 10^−14^) for the *YAP1* transcription factor (expressed by 79% of SCPs; Figure 8A).

To determine which developmental stage described by Furlan et al. [53] (SCP, bridge cells, chromaffin cells, and sympathoblasts) mostly resembled our *RET* KO clones, we then compared the expression pattern from our *RET* KO clones to these from each individual cell from the Furlan data. Strikingly, the best correlations were found for SCPs (mean Pearson’s *r* = 0.32 for C19 clone) and correlation coefficients decreased over the developmental timeline from bridge cells (mean *r* = 0.20) to chromaffin cells (mean *r* = 0.13). Sympathoblasts, which have been suggested a separate lineage, had intermediate values (mean *r* = 0.22; Figure 8A). The similarity between the *RET* KO NB cells and SCPs was further confirmed by an unsupervised hierarchical clustering analysis on these Pearson correlation coefficients. This analysis demonstrated a clear cluster of highly correlated cells that almost completely recapitulated the SCP classification, on the one hand, and another cluster with weakly correlated cells that largely corresponded to chromaffin cells, on the other hand (Figure 8B). These results indicate that *RET* KO in NB cells promotes mesenchymal identity that closely resembles that of the SCPs during embryonic neural crest development.

### 2.7. RET Is Required for Context-Dependent Differentiation of NB Cells

**Figure 9:**
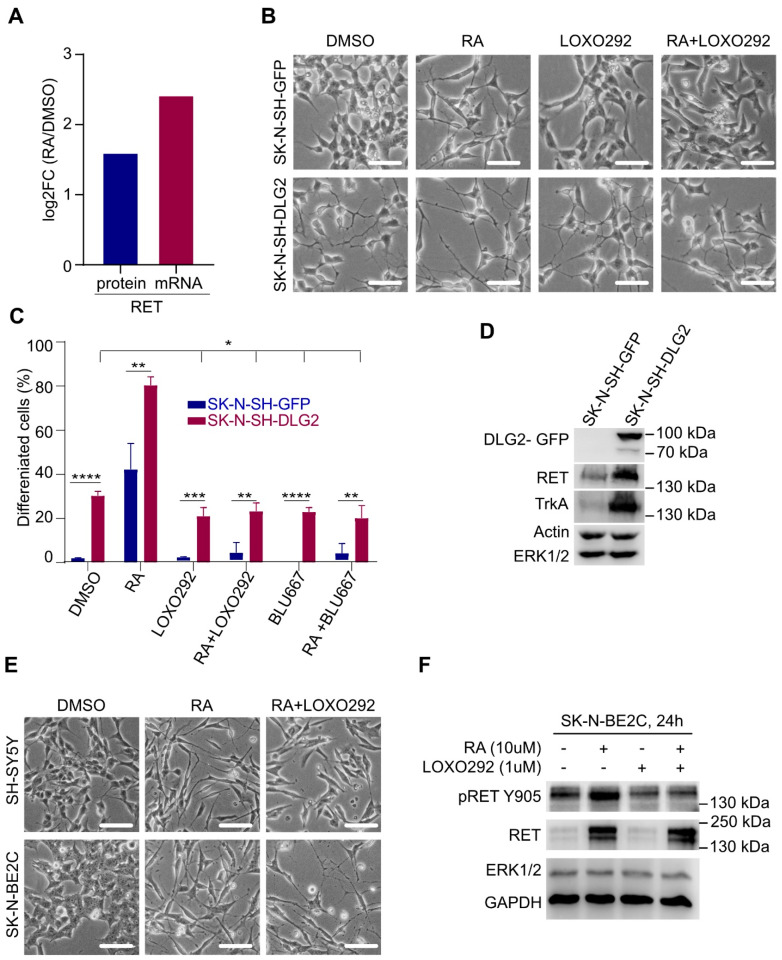
RET is required for context-dependent differentiation of NB cells. (**A**) Bar chart showing the effect of 13-*cis* retinoic acid (RA) on RET protein and mRNA expression from proteomic and RNA-seq data. SH-SY5Y NB cells were treated with either DMSO or RA (10 µM) for 24 h. Proteomic and RNA-seq datasets were previously published by Siaw et al. [43] *p*-adj = 0 for log_2_FC (RA/DMSO) for both RET protein and mRNA. (**B**) Representative images (scale bar = 50 μm) and (**C**) quantification of discs large homolog 2 (DLG2)- or RA-induced morphological differentiation in SK-N-SH-GFP and SK-N-SH-DLG2 NB cells, in the presence and absence of RET inhibitors (LOXO292 and BLU667). * *p*<0.5, ** *p*<0.05, *** *p*<0.005, **** *p*<0.0005. (**D**) Western blot confirming DLG2 overexpression and its effect on expression of neuronal differentiation markers. (**E**) Representative images (scale bar = 50 μm) showing effect of LOXO292 on RA-induced morphological differentiation in SH-SY5Y and SK-N-BE2C NB cell lines. (**F**) Western blot analysis of RET expression and phosphorylation in SK-N-BE2C cells with indicated treatments for 24 h.

Previous data suggested that RET is required for RA-induced differentiation in NB cells [39,44,55,56]. These reports are in alignment with our findings that the expression of *Ret* is enriched in the E13.5 mouse peripheral nervous system at later stages, at which point cells have progressed through the differentiation process (Figure 8A). Analysis of RA-induced RNA-Seq and proteomics data in NB cells [43] shows that both *RET* mRNA and protein levels increase on RA-induced differentiation (Figure 9A). Our observations that RET expression levels correlate with differentiation led us to test the effect of RET TKIs on differentiation of NB cells in a controlled system. To do this we employed discs large homolog 2 (DLG2)-induced and RA-induced differentiation in NB cells, which we previously showed leads to differentiation [43]. Remarkably, treatment with two different RET TKIs (LOXO292 or BLU667) blocked RA-induced differentiation (Figure 9B,C). In contrast, RET inhibition had little effect on DLG2-induced differentiation, despite increased RET protein levels in SK-N-SH-DLG2 NB cells (Figure 9D). The effect of RET inhibition was also tested in two additional NB cells lines that differentiate in response to RA, SH-SY5Y, and SK-N-BE2C. Here, we observed that RET inhibition was able to inhibit RA-induced differentiation in SH-SY5Y cells, but not SK-N-BE2C, even though robust RET protein induction and activation as measured by pRET-Y905 phosphorylation were observed upon RA treatment in SK-N-BE2C (Figure 9E,F). Taken together, our findings suggest that RET activity is important in some NB cell lines for RA-induced differentiation, but it does not appear to be required for DLG2-induced NB cell differentiation.

## 3. Discussion

Our observation that ALK activation in response to ALKAL stimulation of NB cells results in rapid tyrosine phosphorylation of RET suggests coregulation between ALK and RET signaling pathways [27,57]. Interestingly, activation of Trk in sympathetic neuron cultures results in transactivation of Ret [25]. Activation of RET in response to TRK activation has also been shown in NB cells [28,44]. There are additional reports of crosstalk involving heterodimerization and activation among different RTK family members [27,57]. Moreover, such RTK coactivation may be perturbed in cancer cells leading to aberrant regulation of signaling [27].

Elegant work on RET activation dynamics suggests that RET activity is regulated by regions that flank the kinase domain core, rather than phosphorylation of the activation loop, which displays slower phosphorylation kinetics [26]. How this might relate to RET activation in response to the ALK activation observed here or to the TrkB–Ret interplay previously described [28,44] remains to be seen. This rapid modulation of RET tyrosine phosphorylation by ALK is in addition to transcriptional regulation, where ALK signaling was shown to regulate *RET* in NB cell lines and mouse models [21,24,36,37]. Initially, Cazes et al. showed that ALK activation controls *RET* expression in *Th-MYCN/Alk_F1178L* tumors, and they reported upregulation of *RET* expression in human tumors with activated ALK [21]. They also showed that *Th-MYCN/Alk_F1178L* tumors are sensitive to vandetanib, which inhibits RET among other targets [21]. Mutant ALK-driven upregulation of *RET* and RET-driven cholinergic markers was also reported by Lambertz et al., further supporting a rational for testing ALK–RET-oriented molecular combination therapies in NB [24].

In contrast to our findings here, Lambertz and colleagues suggested that regulation of *RET* by ALK was mediated by forkhead box class O 3a (FOXO3a) via PI3K/AKT signaling [24]. Our analyses strongly suggest that ALK signaling regulates *RET* transcription predominantly through the RAS/MAPK signaling arm, and the results are in keeping with later findings investigating ALK and RET in NB [37], with characterization of a *Th-MYCN/RetM919T* mouse model in which oncogenic cooperation between activated Ret and MYCN overexpression resulted in NB that displayed similarities with *Th-MYCN/Alk_F1178L* tumors. Furthermore, a role for ETS variant transcription factor 5 (Etv5) downstream of the RAS/MAPK arm of Alk signaling was shown to be important in the regulation of *Ret* expression [37], in agreement with our findings here. This RAS/MAPK-dependent activation of ETV5 downstream of ALK was independently reported in NB cells [49].

Taken together, several studies have reported ALK regulation of *RET* transcription and some have gone on to propose RET as a bona fide target for molecular treatment of NB [21,24,37]. Our data suggest that the relationship between ALK and RET is complex, involving both transcriptional and post-translational regulation, likely reflecting a delicate interplay of developmental events in the sympathetic nervous system and tumor development that are currently not fully understood. Our results suggest that absence of RET results in a loss of differentiation, with *RET* KO cells exhibiting a strong EMT signature that may represent an undesirable phenotype in clinical terms. Interestingly, acquired resistance to ALK TKI treatment in NB cells was found to be caused by a bypass mechanism involving the increased expression of growth arrest specific 6 (GAS6) and the concomitant activation of its cognate RTK, AXL [58]. This GAS6/AXL-mediated resistance was associated with induction of EMT, with the overexpression of the characteristic mesenchymal markers, SNAI2, FN1, and vimentin (VIM), similar to the EMT phenotype exhibited by the *RET* CRISPR/Cas9 KO cells characterized here. Given these findings, as well as the reports of improved efficacy of response of murine *Th-MYCN/Alk_F1178L* tumors to combined ALK/RET TKI (crizotinib/vandetanib) treatment [37], it is worth considering exploration of TKIs that target both ALK and RET. One such example is alectinib, which was shown to effectively inhibit a range of ALK mutant variants in an NB setting, as well as to effectively inhibit oncogenic RET [59,60].

Gain-of-function *Alk* knock-in mice models were reported to display enlarged sympathetic ganglia, but they did not lead to NB development unless combined with an additional oncogene, such as *Th-MYCN* [20,21,48]. Ret is known to regulate neuronal migration and survival through the sympathetic nervous system [23]. Gain-of-function *Ret* mice were reported that exhibit enlarged sympathetic ganglia (*Ret_M919T*) or increased numbers of enteric neurons (*Ret51_C618F*) [61,62]. Indeed, the *RetM919T* mouse also results in NB tumor development when combined with *Th-MYCN* [37]. Thus, while neither Alk nor Ret activation in mouse models has been shown to be capable of driving NB in the absence of an additional oncogene, aberrant activation of either RTK results in significant disturbance in the development of the sympathetic nervous system.

NB is thought to develop from an aberrantly developed neural crest-derived cell somewhere in vicinity of the adrenal medulla and/or the sympathetic ganglia [63,64]. During development, these cells undergo migration to destinations close to the dorsal aorta where they form the sympathetic ganglia and the chromaffin cells that contribute to the adrenal medulla. Recent work has shed light on the role of the peripheral stem cells known as SCPs during mouse embryonic development [53]. Examination of the E13.5 dataset from this study revealed a striking inverse correlation of *Ret* expression with SCPs, suggesting that expression of *Ret* may be important in the differentiation process. This is agreement with other studies that reported a role for RET in differentiation [23]. More striking, however, was the observation that expression of molecules upregulated in the *RET* CRISPR/Cas9 KO NB cell lines generated in this study was highly correlated with SCPs. This was particularly clear in the case of *Axl* and *Yap*, both of which are heavily expressed in E13.5 mouse SCPs. These observations suggest that *Ret* expression results in a differentiation program that decreases expression of EMT markers and determinants, and this is supported by our findings that reintroduction of RET leads to decreased levels of these molecules. There is a body of evidence supporting a role for RET activity during differentiation; however, the effect of RET TKI treatment on NB cell differentiation appears to be cell-dependent, with RA-induced differentiation of some NB cell lines sensitive to RET TKI treatment, while others are not. This may reflect the complex genetics underlying the various NB cell lines employed here; however, we observe that RA-induced differentiation, i.e., in the SK-N-BE2C NB cell line, proceeds in the presence of LOXO292 despite effective inhibition of pRET-Y905 phosphorylation. Our findings show that RET expression correlates with RA-induced differentiation, but RET activity is not always required for differentiation to occur. Future work addressing RET and ALK receptor activation during this developmental timeline would be helpful to understand the dynamics of their signaling during this process.

## 4. Material and Methods

### 4.1. Antibodies and Inhibitors

The primary antibodies used were anti-pRET Y687 [26] (1:5000; gift from Prof. Neil McDonald, The Francis Crick Institute, UK), anti-pRET Y1062 (1:2000) from Abcam (Cambridge, UK), and anti-RET (E1N8X, 1:2000), anti-pRET Y905 (1:2000), anti-ALK (D5F3, 1:5000), anti-pALK Y1604 (1:2000), anti-pAKT S473 (1:5000), anti-pERK1/2 (1:2000), anti-FN1 (1:2000), anti-AXL (1:2000), anti-MET (1:2000), anti-YAP (1:2000), anti-GAPDH (1:2000), and anti-β actin (1:2000), all from Cell Signaling Technology (Danvers, MA, USA). The primary antibodies used for immunoprecipitation were anti-RET (C31B4, 1:50, Cell Signaling Technology, Danvers, MA, USA), anti-ALK mAb53 (generated as described in [65], experimentally validated in Figure S1B), and mouse immunoglobulin G (IgG) control (Santa Cruz Biotechnology, Dallas, TX, USA). The secondary antibodies were goat anti-mouse IgG horse radish peroxidase (HRP) conjugate and goat anti-rabbit IgG HRP conjugate purchased from Invitrogen Antibodies (Thermo Fisher Scientific, Waltham, USA). The ALK inhibitor crizotinib and RET inhibitors LOXO292 and BLU667 were purchased from MedChemtronica AB (Stockholm, Sweden). Pan-RAF inhibitor LY3009120, MEK1/2 inhibitor trametinib, ERK1/2 inhibitor SCH772984, RSK1/2/3/4 inhibitor BI-D1870, AKT1/2/3 inhibitor MK-2206, S6K1 inhibitor PF-4708671, and mTOR inhibitor rapamycin were purchased from Selleckchem (Houston, TX, USA). Recombinant ALKAL2 was produced by IBA Lifesciences (Göttingen, Germany). Recombinant human glial-derived neurotrophic factor (GDNF), retinoic acid (R2625), doxycycline (D1822), and cycloheximide (01810) were purchased from Sigma-Aldrich of Merck Group (Darmstadt, Germany). Protein G Sepharose 4 Fast Flow (17-0618-01) was purchased from GE Healthcare Bio-Sciences AB (Uppsala, Sweden).

### 4.2. Cell Culture and Cell Treatments

Neuroblastoma cell lines NB1 (ALK-amplified), CLB-GE (ALK-F1174V mutant), SK-N-AS (wildtype ALK but no expression, RAS-Q61K mutant), CLB-BAR (Δexon4–11 truncated ALK, ALK-amplified), CLB-GAR (ALK-R1275Q mutant), IMR-32 (wild-type ALK), Kelly (ALK-F1174L mutant), SK-N-BE2C (wildtype ALK), SK-N-DZ (wild-type ALK), SH-SY5Y (ALK-F1174L mutant), and SK-N-SH (ALK-F1174L mutant) were cultured in complete medium, RPMI 1640 supplemented with 10% fetal bovine serum (FBS) and a mixture of 1% penicillin/streptomycin at 37 °C and 5% CO_2_.

ALK-inducible SK-N-AS (ALK-teton) cells were generated as described below and cultured as above. Recombinant ALK (GSATx4 linker-BirAR118G-HA) was ordered from IDT as a gBlock (IDT, Coralville, USA) and cloned into pcDNA3.1-ALK (NM_004304.3) using NEBuilder HiFi DNA Assembly (NEB, Ipswich, USA). ALK-GSATx4-BirAR118G was then subcloned into the pLVX-TRE3G (Takara, Kusatsu, Japan) lentiviral vector by using infusion HD cloning (Takara, Kusatsu, Japan) to generate pLVX-TRE3G-ALK-GSATx4-BirAR118G. Lentivirus was produced in HEK293T cells using Xfect Transfection reagent and Lenti-X lentiviral packaging plasmids (Takara, Kusatsu, Japan) according to the manufacturer’s protocol. SK-N-AS cells were transduced with lentivirus supernatant, and selection with 1 µg/mL puromycin was used to establish inducible cell lines.

To verify the phosphorylation of RET upon ALK activation and their interaction, NB1 cells or SK-N-AS (ALK-teton) cells were seeded in collagen-coated 6 cm dishes. To induce the expression of ALK, 1 μg/mL of doxycycline was added to the SK-N-AS (ALK-teton) cells 24 h prior to ligand stimulation. The cells were stimulated with 0.5 μg/mL of ALKAL2 or 100 ng/mL of GDNF for 10 min. For stimulation combined with inhibitors, cells were pretreated with crizotinib (250 nM) or LOXO292 (1 μM) for 2 h.

To validate the regulation of RET expression by ALK activity, ALK-driven CLB-GE and NB1 cells were treated with 250 nM of crizotinib for 24 h prior to analysis. To check the protein stability of RET, SK-N-AS cells were treated with 20 μM of cycloheximide (CHX) for time-points indicated in the figure. To test which signaling pathway regulates RET expression, SK-N-AS cells and CLB-GE cells were treated with inhibitors targeting the RAS/MAPK/RSK pathway and PI3K/AKT/mTOR pathway indicated in the figure for 24 h. Cells were harvested and lysed immediately after treatment.

### 4.3. Plasmids, siRNA, and Cell Transfection

The pcDNA3.1+/*RET* (NM_020975.4) plasmid was purchased from GenScript (Piscataway, USA) and was introduced into cells using Lipofectamine 3000 according to manufacturer’s protocol. Cells transfected with empty pcDNA3 vector were used as control. RET siRNAs were Ambion Silencer Select Predesigned siRNA purchased from ThermoScientific (Waltham, MA, USA). They were siRNA1 (identifier (ID): s11935) targeting GCTTGTCCCGAGATGTTTA, siRNA2 (ID: s11936) targeting GGATTGAAAACAAACTCTA, siRNA3 (ID: s11937) targeting CCACTGCTACCACAAGTTT, and siRNA4 (ID: s531214) targeting CACATGTCATCAAATTGTA. SK-N-AS cells were transfected with siRNAs using Lipofectamine RNAiMAX transfection reagent (Thermo Fisher Scientific, Waltham, MA, USA) according to the manufacturer’s protocols. Cells transfected with scrambled siRNA were used as negative controls. Four days after siRNA transfection, one-third of the cells were replated into new wells and subjected to another round of transfection, and the remaining cells were harvested for further analysis.

### 4.4. Cell Lysis, Immunoprecipitation, and Western Blot Analysis

Treated cells were directly lysed in 1× SDS sample buffer. For immunoprecipitation, cells were lysed in 0.5 mL of lysis buffer (50 mM Tris-Cl, pH7.4, 250 mM NaCl, 1 mM EDTA, 1 mM EGTA, 0.5% Triton X-100, complete protease inhibitor cocktail, and PhosSTOP phosphatase inhibitor cocktail) on ice for 20 min prior to clarification by centrifugation at 14,000 rpm at 4 °C for 15 min. Then, 30 μL of supernatant was taken and used as input control, and the remaining sample was incubated with 10 μL of anti-RET antibody, 2 μg of anti-ALK mAb53 antibody, or 2 μg of mouse IgG control for experimental purposes for 2 h at 4 °C. Then, 20 μL of protein G Sepharose beads were added and further incubated at 4 °C overnight. The beads were then washed five times with lysis buffer and boiled in 80 μL of 1× SDS sample buffer. Both inputs and immunoprecipitation products were separated by 9% SDS-PAGE gel and blotted with antibodies indicated in the figures.

Precleared lysates were separated on SDS-PAGE, transferred to polyvinylidene difluoride membranes (Millipore, Burlington, VT, USA), blocked in 3% bovine serum albumin (BSA), and immunoblotted with primary antibodies overnight at 4 °C. Secondary antibodies were diluted 1:20,000 and incubated with shaking at room temperature for 1 h. SuperSignal West Pico PLUS chemiluminescent substrates were used for detection (ThermoScientific, Waltham, USA), and membranes were scanned using the LI-COR Odyssey Fc imaging system (LI-COR Biosciences, Lincoln, NE, USA).

### 4.5. Generation of CRISPR/Cas9 Engineered RET KO SK-N-AS Cells

ThermoScientific CRISPR794433_CR guide RNA (gRNA), which targets the DNA sequence (GGCTCCGGTTAAGGTAGAGG) on the reverse strand of *RET* gene, was used to generate *RET* KO from the parental SK-N-AS cell line (ECACC 94092302, Merck, Darmstadt, Germany). Transfection was performed with Lipofectamine CRISPRMAX Cas9 transfection reagents (ThermoScientific, Waltham, MA, USA) according to the manufacturer’s protocol. Forty-eight hours after transfection, cells were harvested for Western blot analysis to confirm the KO efficiency. Parallel cell samples were harvested and serially diluted to 8–10 cells/mL. The resulting cell suspension was subsequently seeded into 96-well plates at 100 μL per well. The resulting single-cell clones were expanded and subjected to Western blot analysis to confirm loss of RET protein. Five *RET* KO clones and one clone with higher RET expression level were chosen, and, together with parental cells, they were subjected to RNAseq analysis (Novogene, Cambridge, UK) and Cell Line Authentication test (Eurofins Genomics, Ebersberg, Germany).

### 4.6. Cell Proliferation Assay

Five thousand cells of the various *RET* KO and control SK-N-AS clones were seeded into 96-well plates in triplicate. After cell attachment, medium was replaced with fresh medium containing Nuclight Rapid Red Dye for live-cell nuclear labeling (Essen BioScience, Ann Arbor, MI, USA) to label cell nuclei. A Sartorius Incucyte S3 Live-Cell Analysis System (Essen BioScience, Ann Arbor, MI, USA) was used to monitor cell growth and proliferation every 6 h over a time course of 90 h. Proliferation curves were generated by normalizing the red object (nuclei) count per image to 0 h.

### 4.7. Cell Migration Assay

The cell migration assay was performed with Radius 24-Well Cell Migration Assay Kit (Cell Biolabs, San Diego, CA, USA) according to the manufacturer’s protocol. Due to the cell size difference of the SK-N-AS-derived *RET* KO clones and controls, 6 × 10^5^ parental cells, 8 × 10^5^ C1 cells, and 2 × 10^5^ other *RET* KO clone cells were seeded per well. Phase-contrast images were taken at indicated time-points and were analyzed with ImageJ 1.53a software.

### 4.8. Differentiation Assay

Stable GFP- or DLG2-GFP-overexpressing NB cells (SK-N-SH-GFP or SK-N-SH-DLG2) were established as described by Siaw et al. [43]. Cells (5 × 10^4^ cells/well) were seeded in six-well plates and cultured overnight in RPMI-1640, supplemented with 1% penicillin/streptomycin and 10% heat-inactivated FBS. Cells were then treated, where indicated, with 250 nM concentration of the RET inhibitors (RETi), i.e., LOXO292 or BLU667 for 24 h prior to treatment with retinoic acid (RA). After this, cells were treated with RA (10 µM), LOXO292 (250 nM), BLU667 (250 nM), or combination of RA and a RETi in “differentiation medium” (composed of 1:1 mixture of Dulbecco’s modified Eagle’s medium and F12 medium supplemented with 1% heat-inactivated FBS, 2 mM l-glutamine, and 1% penicillin/streptomycin). Cells were scored for differentiation after 48 h of RA treatment. Cells were considered as differentiated when a neurite projecting from a cell body was at least 1.5 times the size of the cell body. At least 200 cells were counted in at least four random fields under microscope, and the number of differentiated cells was noted and calculated for percentage differentiation, while graphs were plotted with Graphpad Prism 9.0. Differentiated cells were photographed using an Olympus CK40 microscope mounted with Olympus DP12 camera (Olympus Optical, Tokyo, Japan). Results are mean of three independent biological replicates. Two additional NB cell lines, SH-SY5Y and SK-N-BE2C, were treated with 10 μM RA in the absence or presence of 1 μM LOXO292. Cells treated with dimethyl sulfoxide (DMSO) were used as mock control. Forty-eight hours later, cells were examined and imaged under bright-field microscope.

### 4.9. RNA-Seq Data Analysis

RNA-seq paired-end reads (read length 150 base pairs) were aligned to the human GRCh38 reference genome using hisat2 [66]. The average alignment efficiency was 94%. Genes were annotated using GENCODE 29 [67] and quantified using featureCounts [68]. Only coding genes were used for further analysis. Differential gene expression was determined using DESeq2 [69]. Genes were considered differentially expressed if their absolute log_2_ fold-change values were >2 at FDR-adjusted *p*-values < 0.01.

For ADRN and MES score analysis, signature genes were derived from van Groningen et al. [45]. Within each sample, the rank percentile of an individual gene’s expression value was used to define a gene score. The ADRN and MES gene signature score was then defined as the mean gene score of all ADRN and MES signature genes, respectively.

### 4.10. Proteomic Data Analysis

Global quantitative analyses of differentially expressed proteins using tandem mass tag (TMT) and isobaric labeling were performed at the Proteomics Core Facility of the University of Gothenburg. The data files for the TMT set were merged for identification and relative quantification using Proteome Discoverer version 2.2 (ThermoScientific, Waltham, MA, USA). The search was performed by matching against the *Homo sapiens* Swissprot Database (version November 2017, Swiss Institute of Bioinformatics, Lausanne, Switzerland) using Mascot 2.5 (Matrix Science, Boston, MA, USA) with a precursor mass tolerance of 5 ppm and fragment mass tolerance of 0.6 Da. Tryptic peptides were accepted with zero missed cleavage, variable modifications of methionine oxidation, and fixed cysteine alkylation; TMT-label modifications of N-terminal and lysine were selected. Percolator was used for the validation of identified proteins, and the quantified proteins were filtered at 1% FDR and grouped by sharing the same sequences to minimize redundancy. TMT reporter ions were identified in the MS3 high energy collision dissociation (HCD) spectra with 3 mmu mass tolerance, and the TMT reporter intensity values for each sample were normalized on the total peptide amount. Only peptides unique for a given protein were considered for quantification. Differential protein expression was determined using the R *ROTS* package [70].

### 4.11. Gene Set Enrichment Analysis

Pre-ranked gene set enrichment analysis (GSEA) was performed using the R *fgsea* package 1.16.0 (*fgseaMultilevel* function, default parameters) with ranking based on the absolute value of the DEseq2 statistic (RNA-Seq) or *ROTS* statistic (proteomics). Hallmark gene sets sere downloaded from the Molecular Signatures Database v7.2 [71].

### 4.12. Correlation Analysis between RET KO Clones and Single-Cell Data

Single-cell gene expression data from Furlan et al. (stage E13.5) were accessed through Gene Expression Omnibus (GEO) (GSE99933) [53]. Sample information and clustering results (classification in SCP, bridge, chromaffin or sympathoblast cells) were derived from http://pklab.med.harvard.edu/cgi-bin/R/rook/nc.SS2_16_250-2/index.html accessed on 13 December 2020. The downloaded gene count data were normalized using DEseq2. Mouse Mouse Genome Informatics (MGI) symbols were converted to human HUGO Gene Nomenclature Committee (HGNC) symbols. A Pearson correlation analysis was then performed between each clone and each cell line. Mutual exclusivity between *Ret* and *Axl* was determined using Fisher’s exact test after binarization of the normalized expression data (i.e., 0 counts = non-expressed, >0 = expressed). Unsupervised hierarchical clustering analysis was performed on the correlation coefficients, and heatmaps were generated using the R *gplots* package 3.3.1. Clustering was performed using the Manhattan distance function.

## 5. Conclusions

Together, our results highlight a crosstalk between the ALK and RET RTKs in NB cell lines. This is in addition to the ALK-driven regulation of RET expression at the transcriptional level reported earlier. We further show that NB cells, in which RET has been genetically ablated, display a less differentiated phenotype and a robust EMT signature at both the mRNA and the protein level. *RET* CRISPR/Cas9 KO cells display increased levels of AXL and MET RTKs, as well as YAP and a number of ECM components. They also display increased migratory activity in agreement with the observed EMT signature, suggesting that inhibition of RET alone in NB may promote a less differentiated phenotype. In summary, our findings suggest that the role of ALK and RET in NB tumor formation is complex; however, dual inhibition of these two RTKs may be beneficial in ALK-driven NB.

## Data Availability

The mass spectrometry proteomics data were deposited to the ProteomeXchange Consortium via the PRIDE partner repository with the dataset identifier PXD024551. The RNA-seq data were deposited (ArrayExpress, https://www.ebi.ac.uk/arrayexpress/; accessed on 14 January 2021. accession no. E-MTAB-10032). All other data required to evaluate the conclusions in the paper are in the paper or Supplementary Materials.

## Supplementary Materials

The following are available online at https://www.mdpi.com/article/10.3390/cancers13081909/s1, Table S1. RNA-Seq and proteomic results. Table S2. RET gene indel status of different SK-N-AS clones from RNAseq data. Figure S1. Experimental validation of ALK and RET antibodies in NB cells. Figure S2. Effect of RET stimulation with GDNF in different ALK-driven NB cells. Figure S3. Multinucleated RET-KO cells. Bright-filed microscopy image to show the multinucleated cells in RET-KO clones after a few passages. Figure S4. Differential protein expression analysis of RET KO NB cell lines.
