# Supplementary material for: Loss of RET Promotes Mesenchymal Identity in Neuroblastoma Cells
Source: Cancers (Basel). 2021 Apr 15;13(8):1909. doi: 10.3390/cancers13081909 (PMC8071449; doi:10.3390/cancers13081909)

Loss of RET promotes mesenchymal identity of neuroblastoma cells

Original images

Fig 1-A

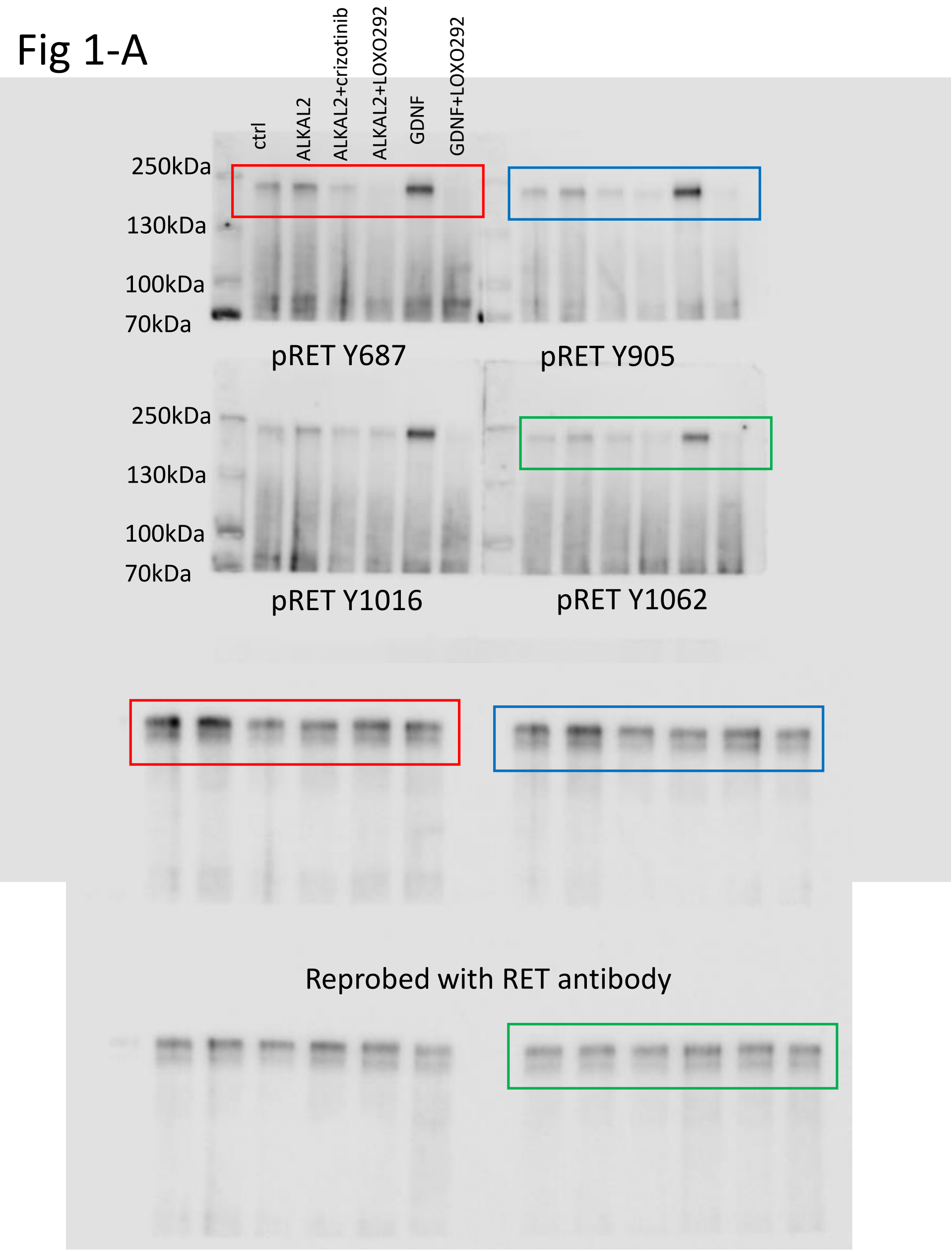

Fig 1-B

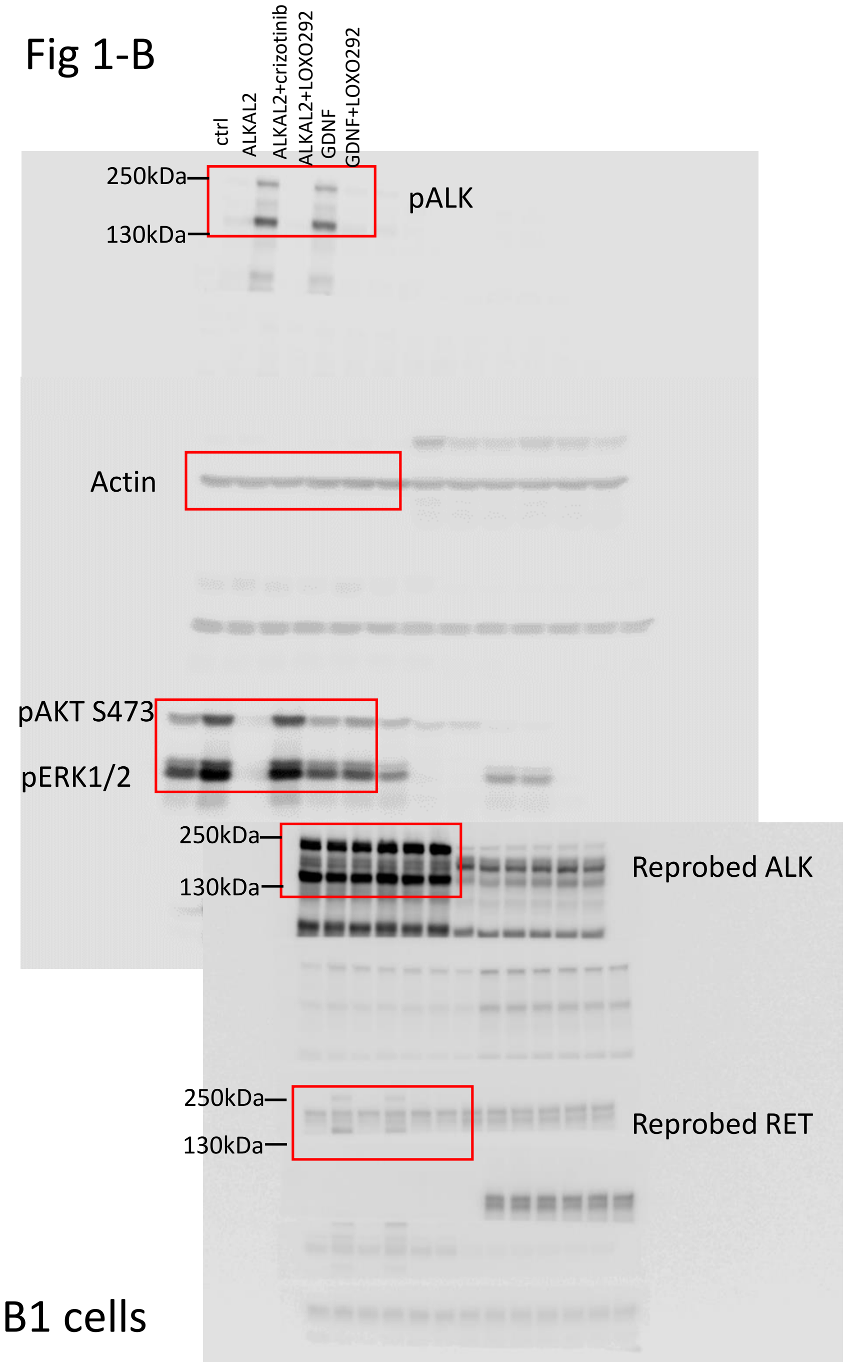

NB1 cells

Fig 1C

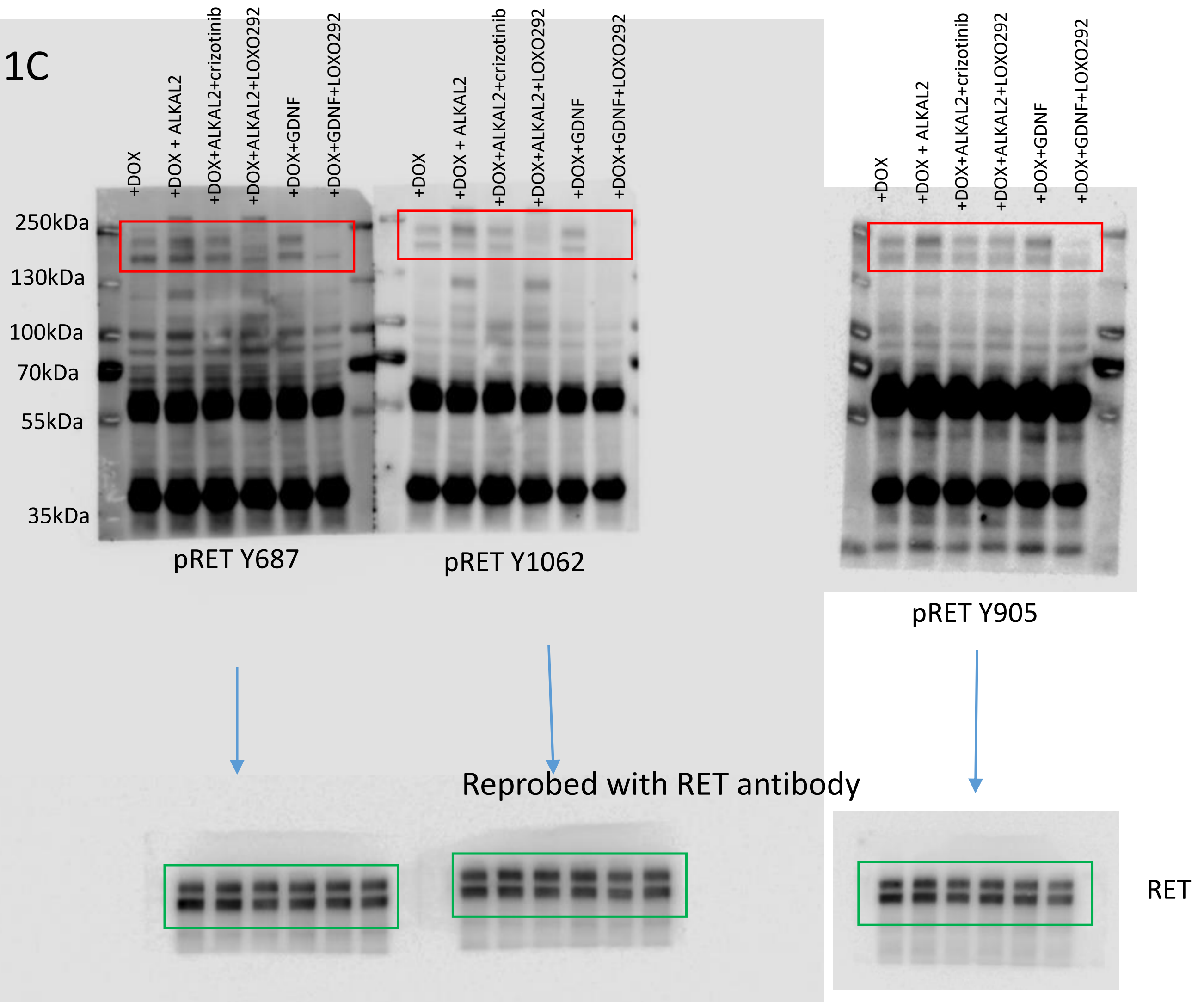

Fig 1D

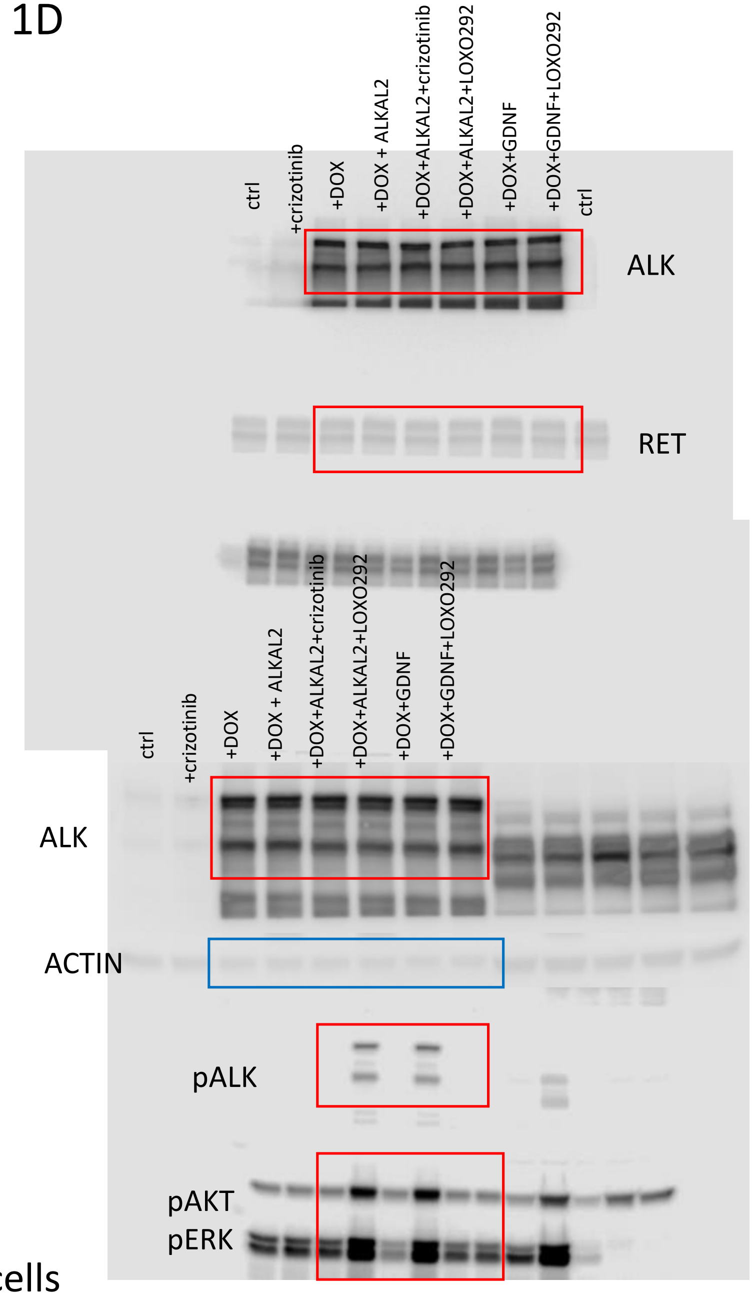

SK-N-AS (ALK teton) cells

Fig. 2A-B ALK IP RET in NB1 cells.

Fig. 2A

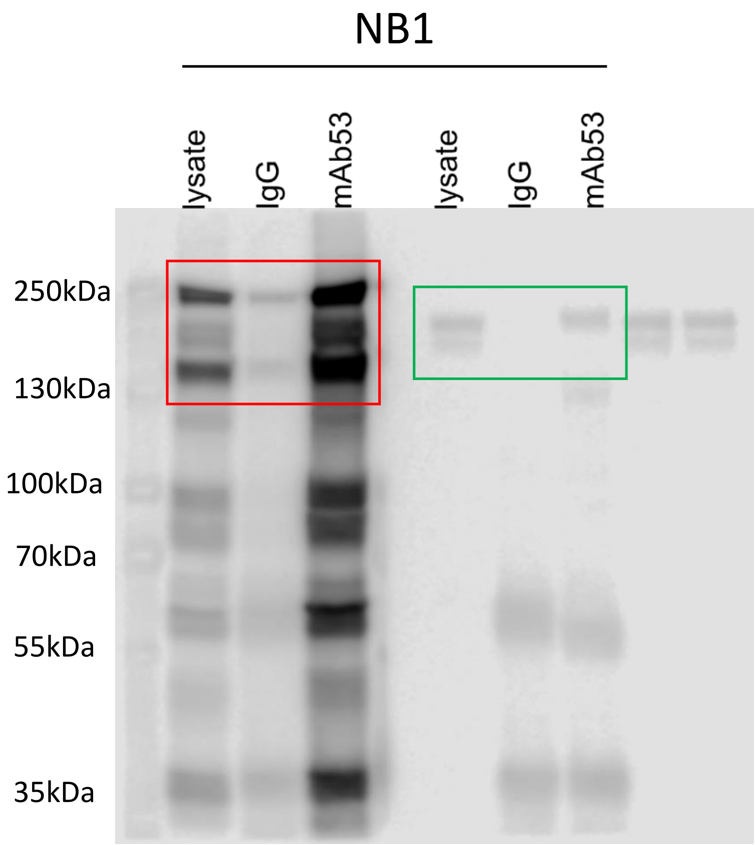

Fig. 2B

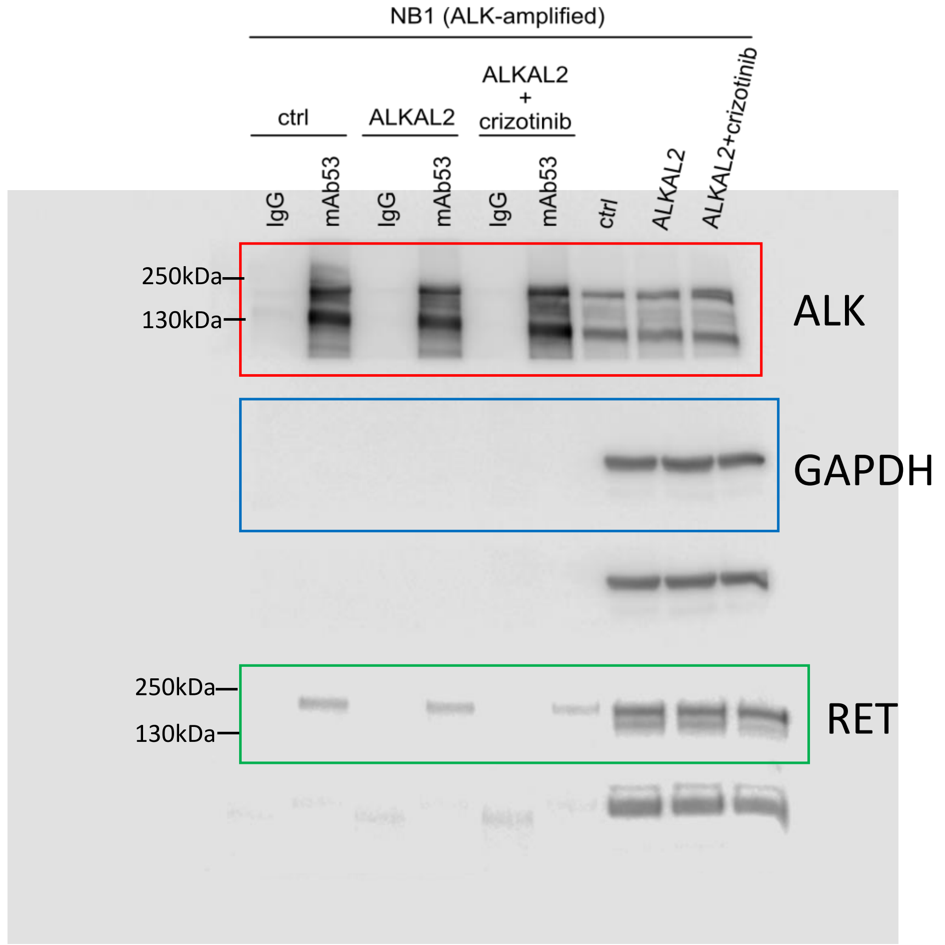

Red box: ALK blot used in main figure
Green box: RET blot
Blue blox: GAPDH control

Fig. 2C-ALK IP RET in SK-N-AS (ALK teton) cells

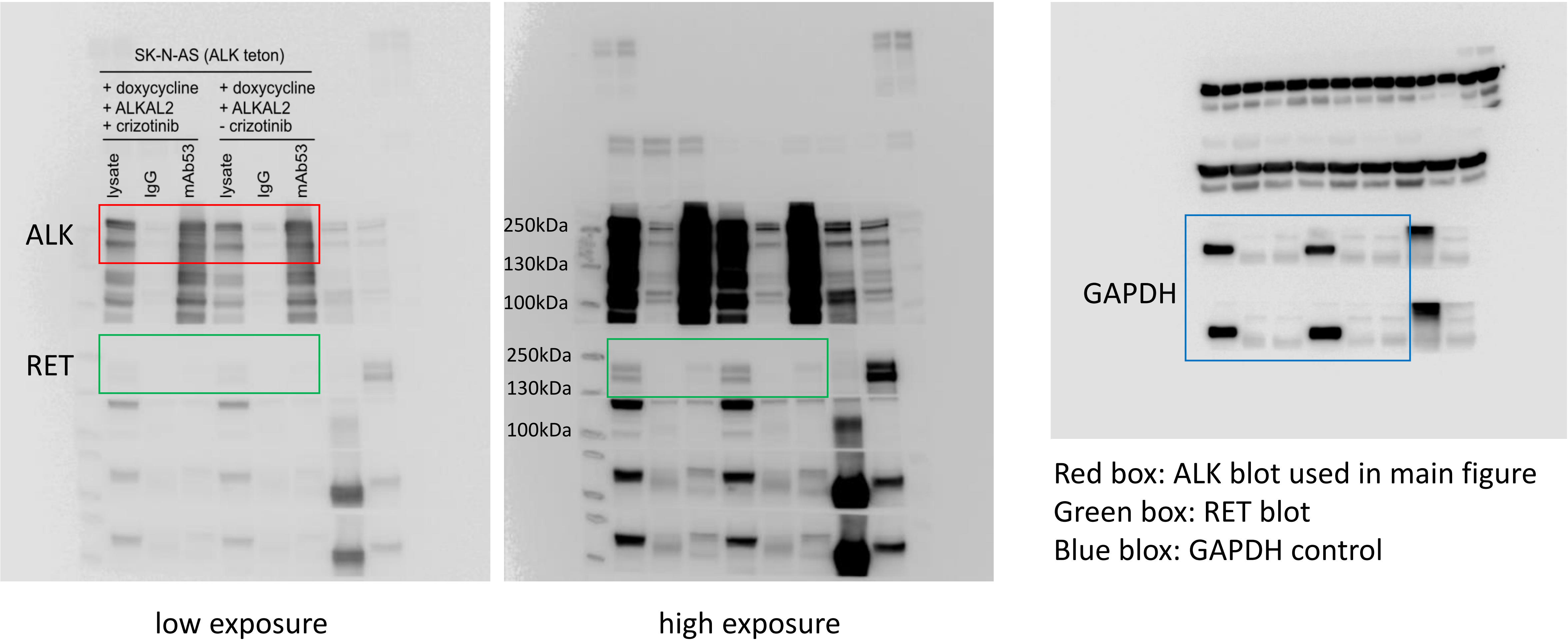

Red box: ALK blot used in main figure
Green box: RET blot
Blue blox: GAPDH control

Fig 3A

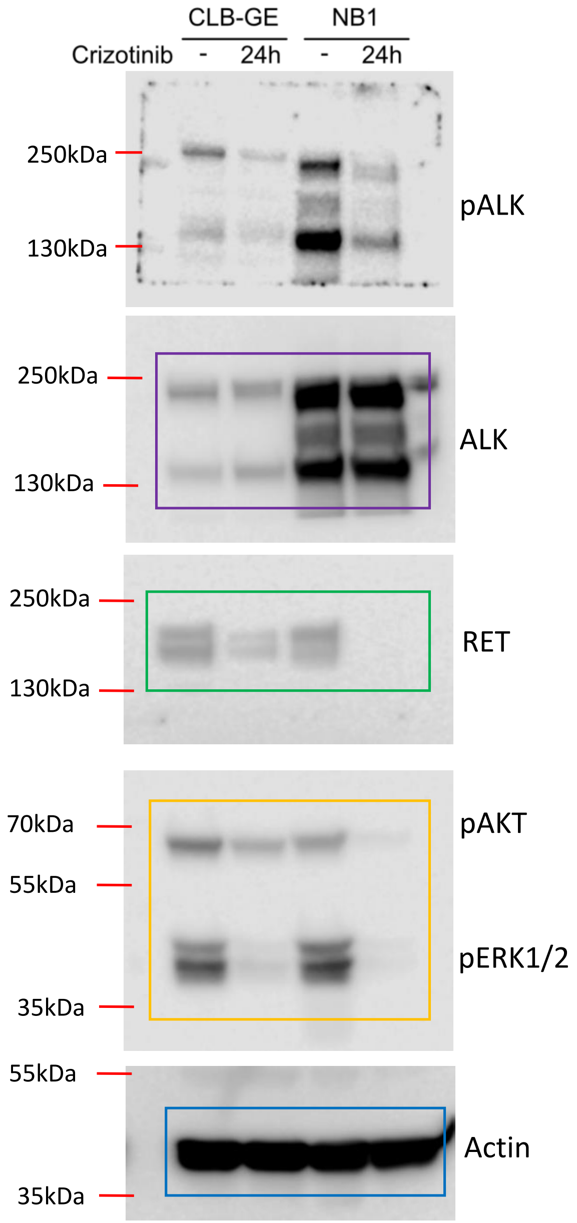

Fig 3B

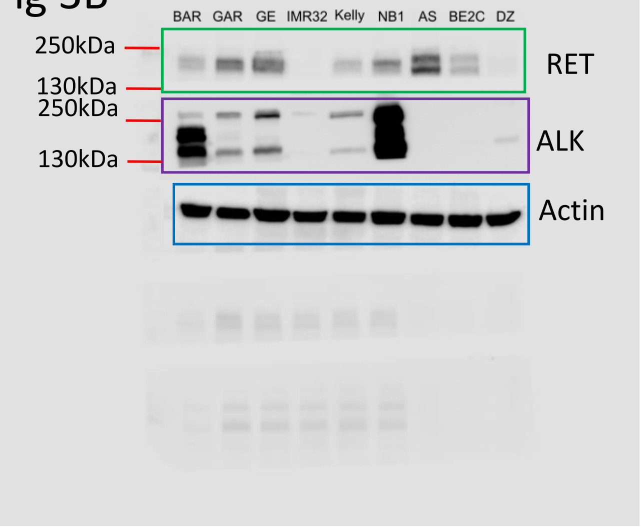

Fig 3C

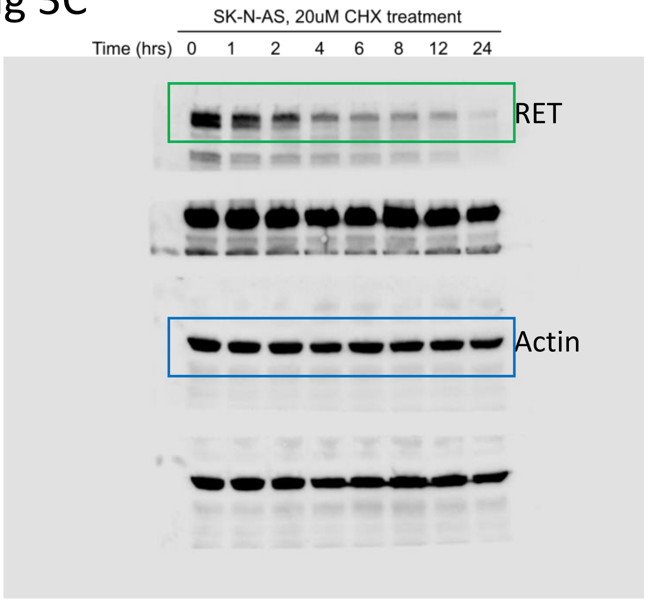

Fig 3D

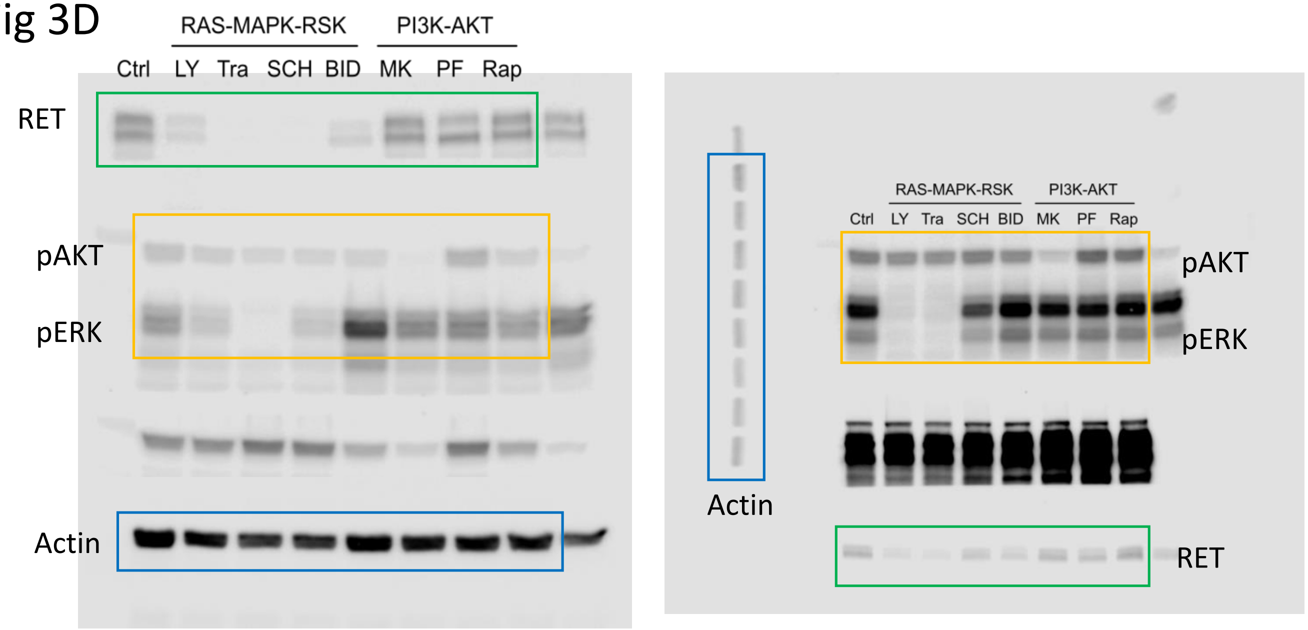

Fig. 3E-time course of treatment with tra and LY

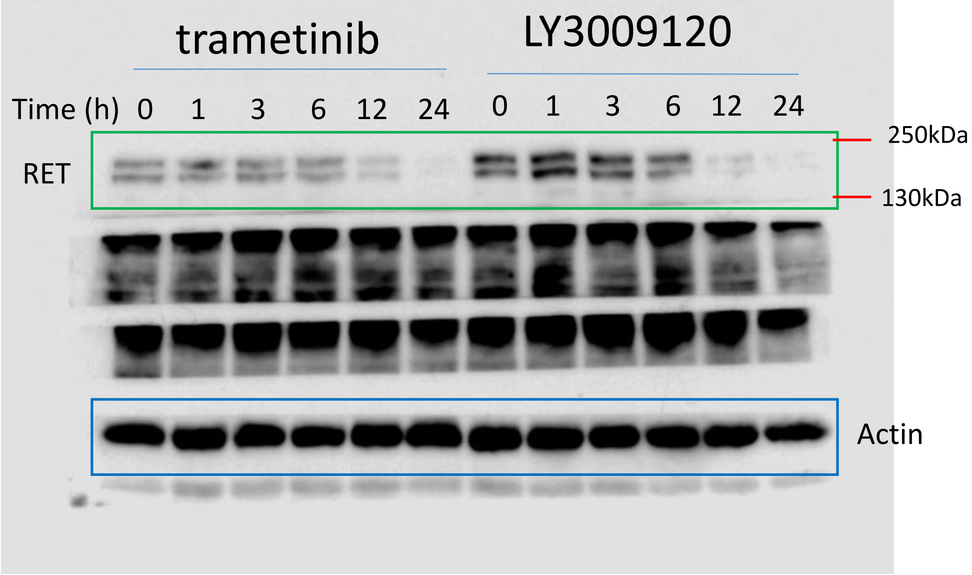

SK-N-AS

Fig. 4A-confirmation of RET expression in different clones

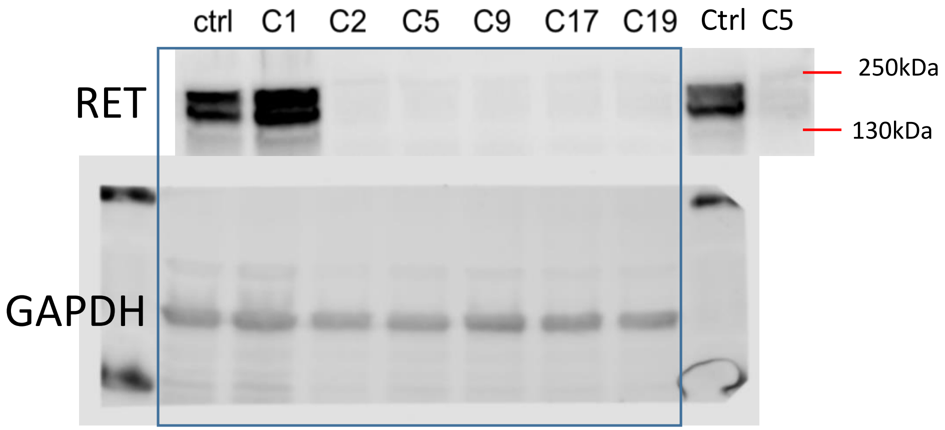

Fig. 7A

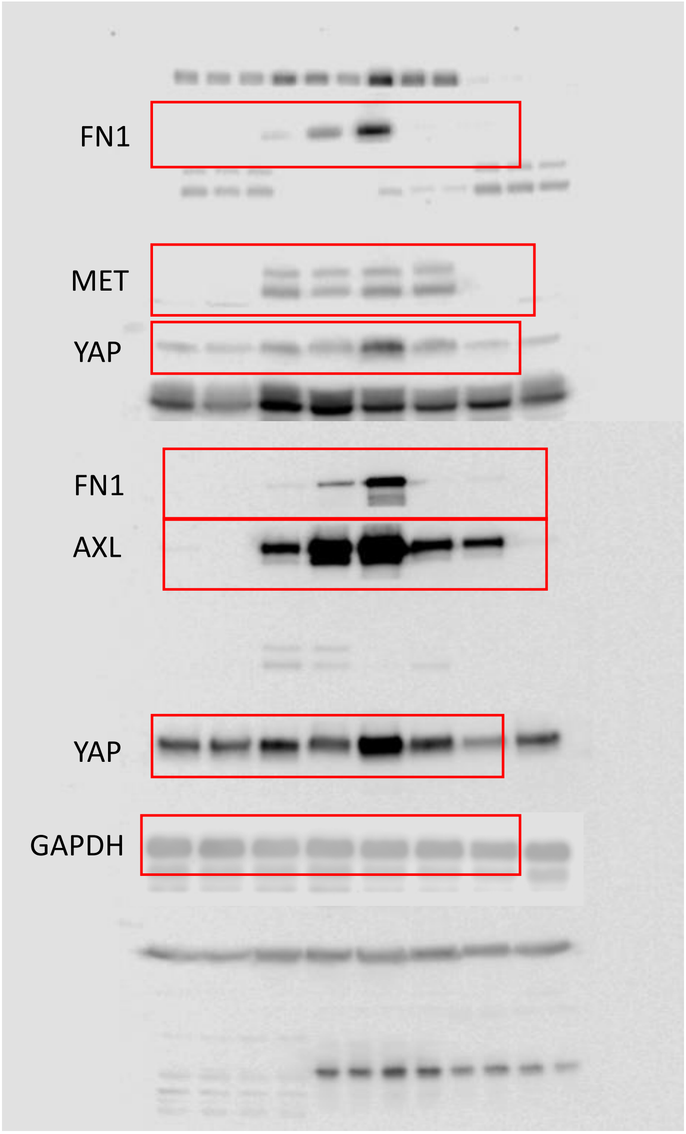

Fig. 7B

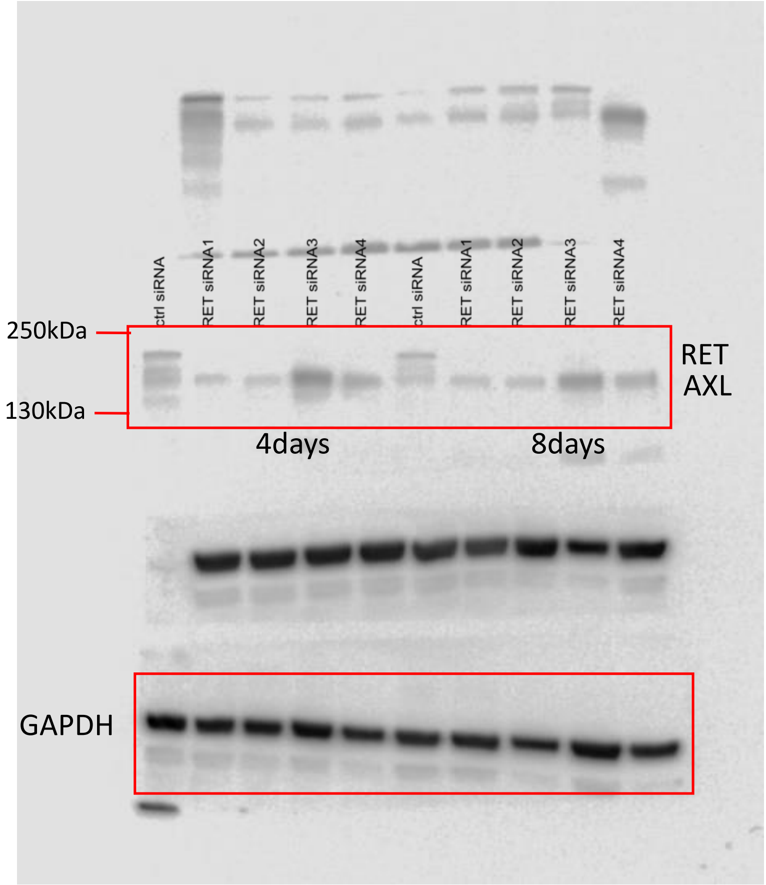

Fig. 7C

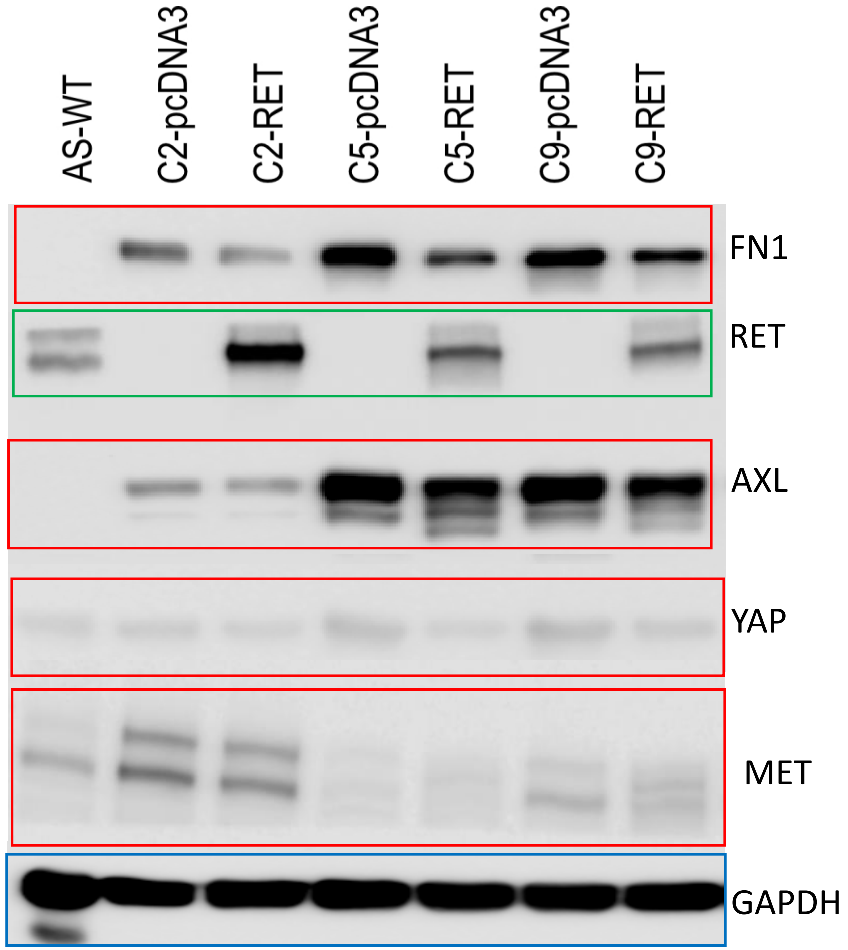

Fig. 9D

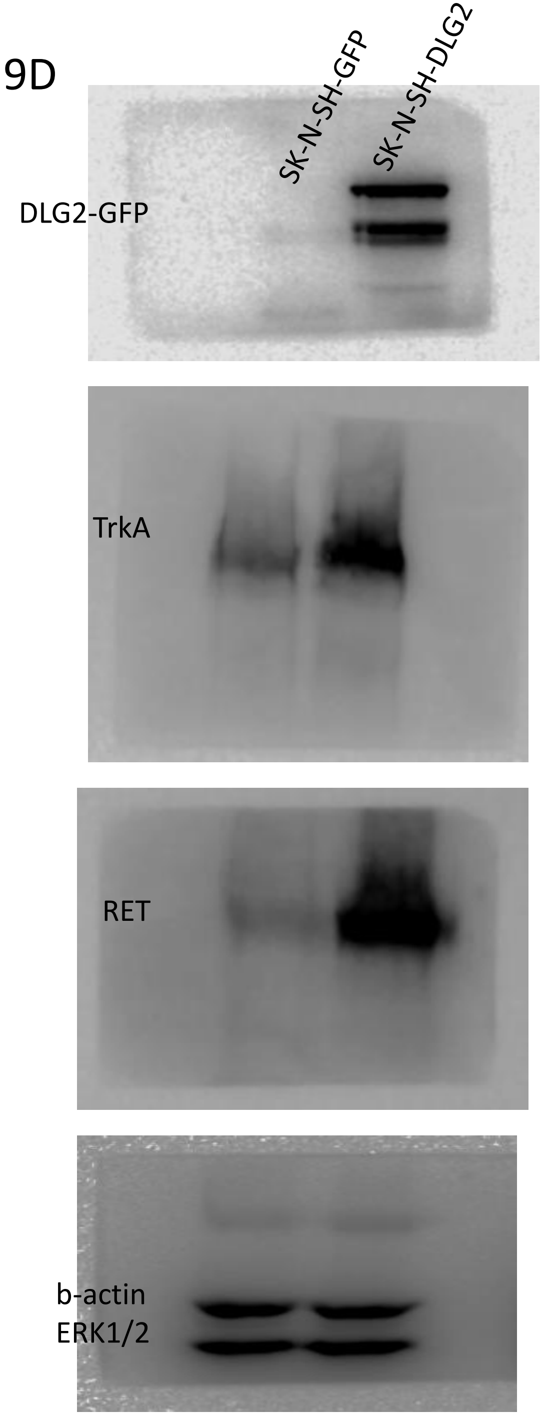
SK-N-SH-GFP
SK-N-SH-DLG2
DLG2-GFP
TrkA
RET
b-actin
ERK1/2

Fig. 9F

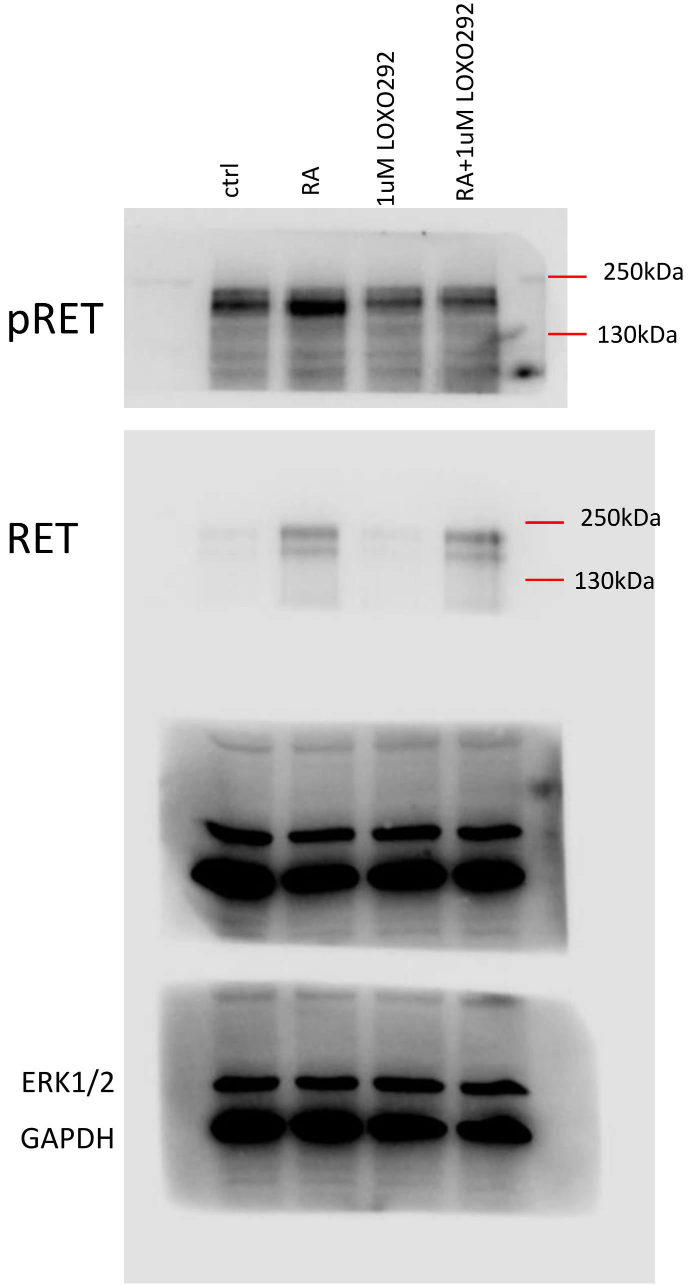
ctrl
RA
1uM LOXO292
RA+1uM LOXO292
pRET
250kDa
130kDa
RET
250kDa
130kDa
ERK1/2
GAPDH

Fig S1-A: Cross-reaction of pRET antibodies to phosphorylated ALK

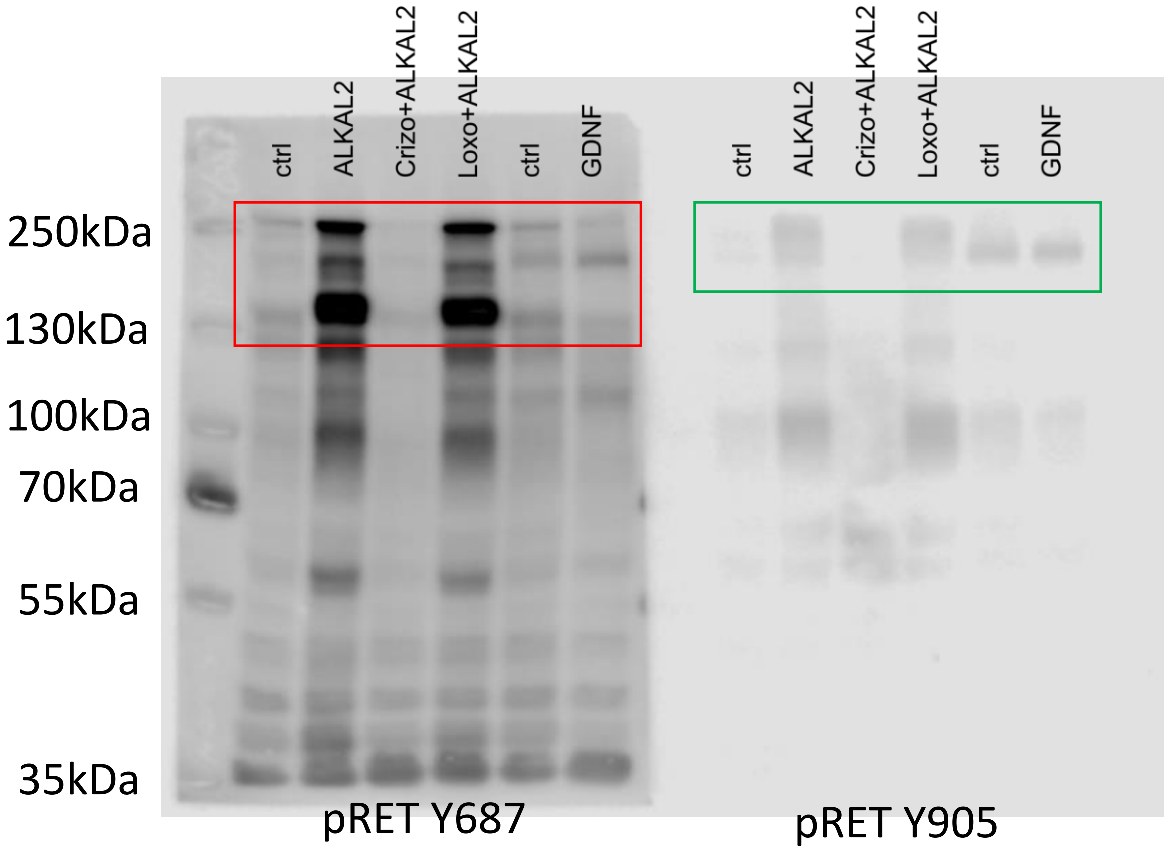

Due to hyperactivation of ALK upon stimulation with its ligand ALKAL2, pALK can also be detected with pRET antibodies. To avoid this cross-reaction, RET IP was performed to pull down total RET and then blot with pRET (see figure 1).

Fig. S1B: test ALK monoclonal antibody mAb53. The use of this antibody has not been published somewhere, so here to verify it.

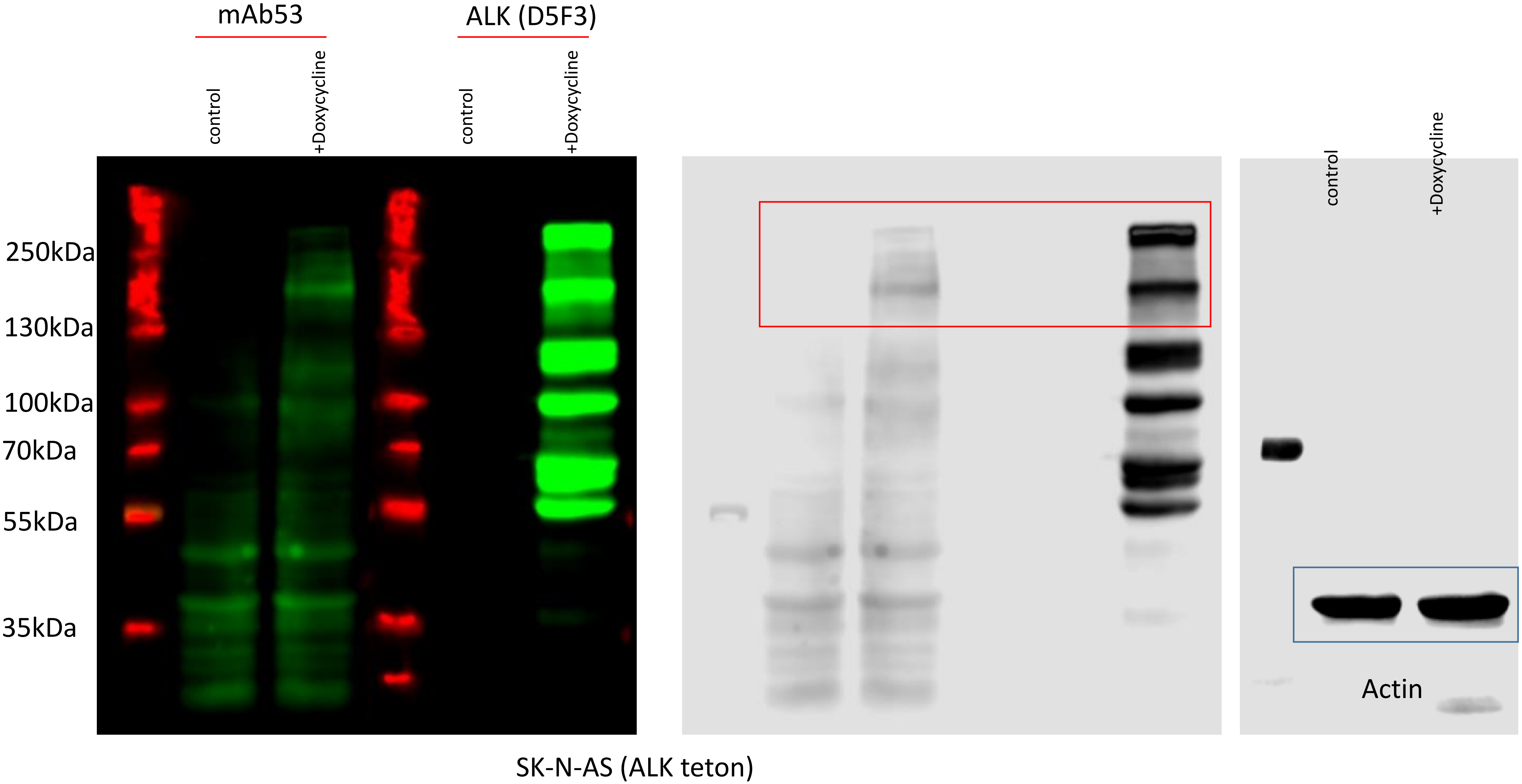

Fig S2. RET stimulation or inhibition in different ALK-driven NB cell lines.

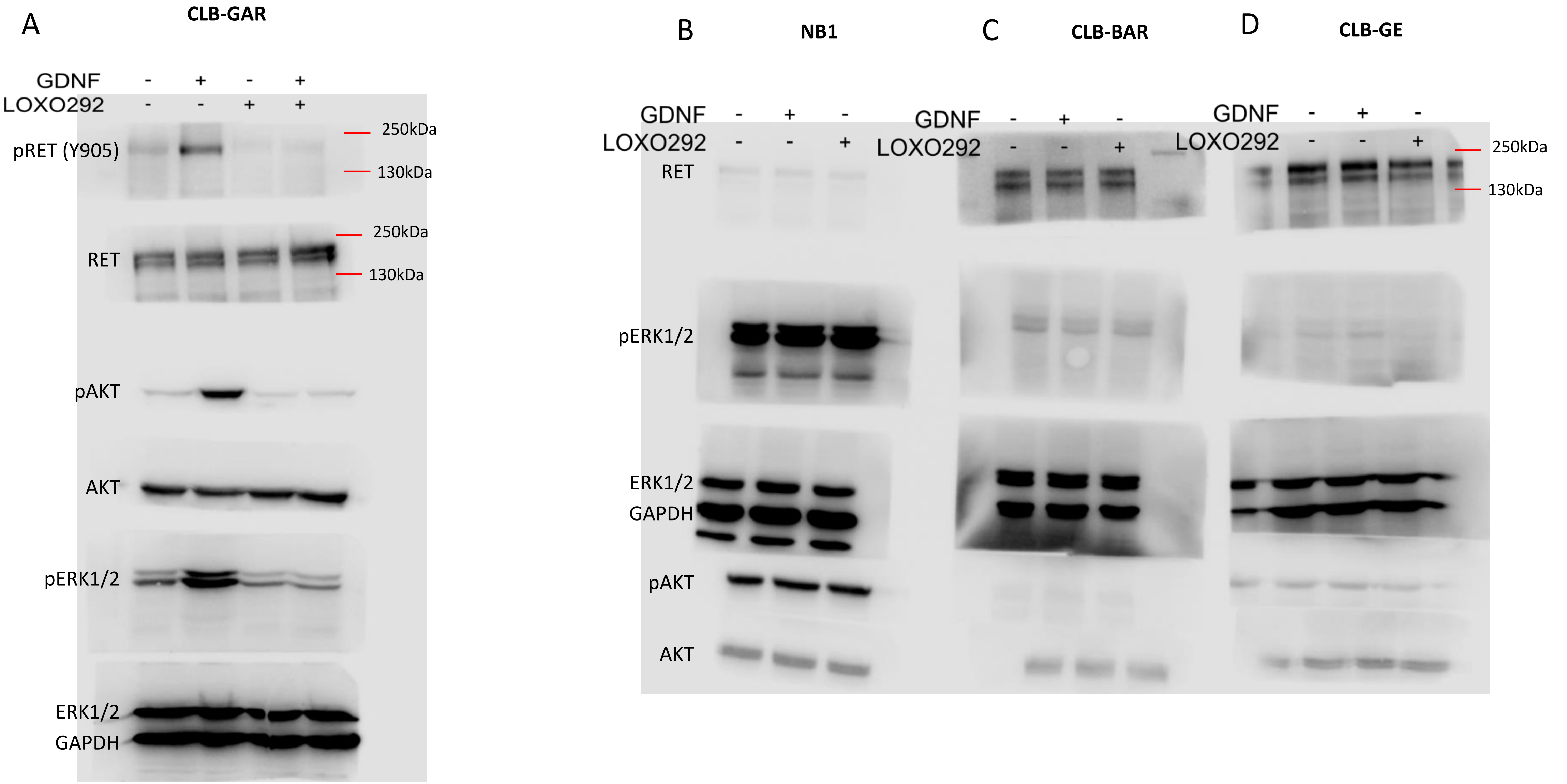

Supplement: Supplementary file 1 [file cancers-13-01909-s001.zip › cancers-1113255-supplementary.pdf]
